# A General Framework for Thermodynamically Consistent Parameterization and Efficient Sampling of Enzymatic Reactions

**DOI:** 10.1371/journal.pcbi.1004195

**Published:** 2015-04-14

**Authors:** Pedro Saa, Lars K. Nielsen

**Affiliations:** Australian Institute for Bioengineering and Nanotechnology, The University of Queensland, Brisbane, Queensland, Australia; Saint Louis University School of Medicine, UNITED STATES

## Abstract

Kinetic models provide the means to understand and predict the dynamic behaviour of enzymes upon different perturbations. Despite their obvious advantages, classical parameterizations require large amounts of data to fit their parameters. Particularly, enzymes displaying complex reaction and regulatory (allosteric) mechanisms require a great number of parameters and are therefore often represented by approximate formulae, thereby facilitating the fitting but ignoring many real kinetic behaviours. Here, we show that full exploration of the plausible kinetic space for any enzyme can be achieved using sampling strategies provided a thermodynamically feasible parameterization is used. To this end, we developed a General Reaction Assembly and Sampling Platform (GRASP) capable of consistently parameterizing and sampling accurate kinetic models using minimal reference data. The former integrates the generalized MWC model and the elementary reaction formalism. By formulating the appropriate thermodynamic constraints, our framework enables parameterization of any oligomeric enzyme kinetics without sacrificing complexity or using simplifying assumptions. This thermodynamically safe parameterization relies on the definition of a reference state upon which feasible parameter sets can be efficiently sampled. Uniform sampling of the kinetics space enabled dissecting enzyme catalysis and revealing the impact of thermodynamics on reaction kinetics. Our analysis distinguished three reaction elasticity regions for common biochemical reactions: a steep linear region (0> *ΔG_r_* >-2 kJ/mol), a transition region (-2> *ΔG_r_* >-20 kJ/mol) and a constant elasticity region (*ΔG_r_* <-20 kJ/mol). We also applied this framework to model more complex kinetic behaviours such as the monomeric cooperativity of the mammalian glucokinase and the ultrasensitive response of the phosphoenolpyruvate carboxylase of *Escherichia coli*. In both cases, our approach described appropriately not only the kinetic behaviour of these enzymes, but it also provided insights about the particular features underpinning the observed kinetics. Overall, this framework will enable systematic parameterization and sampling of enzymatic reactions.

## Author Summary

Kinetic models enable understanding and prediction of the dynamic behaviour of enzymatic reactions. Different frameworks have been proposed to parameterize enzymatic reactions using approximate expressions while maintaining thermodynamic consistency. Approximate expressions have been particularly sought and used, as kinetic expressions typically require large amounts of data to fit their parameters. The latter however ignores real kinetic behaviours and incurs in loss of generality. To overcome these limitations, here we present a novel framework GRASP for exploring the kinetic behaviour of enzymatic reactions under uncertainty based on parameter sampling. By formulating the appropriate thermodynamic constraints and using minimal biochemical reference data, our framework is capable of parameterizing the kinetics of any oligomeric enzyme without sacrificing complexity. To this task, we integrated the generalized MWC model and the elementary reaction formalism, providing a thermodynamically-safe and easy-to-sample parameterization. Application of our framework provided valuable insights into how reactions are regulated under non-equilibrium conditions. We also showed how our approach can be used to describe and understand complex kinetic behaviours of enzymes involved in key regulatory steps of cell metabolism. Overall, this framework enables systematic exploration of the feasible kinetic behaviour of enzymes.

## Introduction

Since the seminal work of Michaelis and Menten [1], enzyme kinetics theory has been developed for most catalytic mechanisms, captured as a series of elementary reactions representing events at molecular level, *i*.*e*. binding and release of reactants from enzyme intermediates and catalysis. The particular catalytic pattern of the enzyme dictates its mathematical representation, which can be obtained by solving equations for the enzyme intermediate concentrations [2]. A quasi-steady-state assumption for these intermediates is commonly employed to this end [3], yielding a final expression function of microscopic rate constants and reactant concentrations. The whole process can be automated using King-Altman’s method [4]. Some of the rate constants are related to the apparent equilibrium constant of the overall reaction by the Haldane relationships, directly linking kinetics with thermodynamics [5]. Moreover the rate constants can be converted into macroscopic kinetic constants [6], which can be measured and estimated from subsequent enzymatic assays.

The classical approach including various graphical representations works well for studying regular enzymes up to a couple of substrates and a couple of products. With more complex reaction mechanisms, the number of parameters becomes so large that a combination of model reduction and/or computational sampling is required for study. Models can be reduced by introducing simplifying assumptions, *e*.*g*., steps being at equilibrium, lumped elementary steps, etc. Multiple expressions have been proposed to reasonably approximate kinetic behaviour using available data while maintaining essential thermodynamic consistency [7–12]; however they always incur some loss of generality. Computational sampling enables us to determine emergent enzyme properties from high dimensional parameter spaces. Several sampling strategies have been proposed, for example uniform parameter sampling within the S-formalism [13,14], elasticity sampling [15,16] and relative enzyme saturation sampling [17,18]. All use simplified kinetic expressions (loss of generality) and most ignore intrinsic thermodynamic constraints between kinetic parameters, hence they will sample infeasible parameter sets.

Current parameterization and sampling approaches also fail to accurately capture allosteric regulation. Even though mass inhibition/activation can account for some degree of regulation, metabolic control is mostly achieved through allosteric and transcriptional regulatory interactions [19]. Modelling allosteric behaviour requires the inclusion of conformational information, which enables description of both allosteric and cooperative interactions. The two most famous of such models are the symmetry model of Monod, Wyman and Changeux (MWC) [20] and the sequential model of Koshland, Némethy and Filmer (KNF) [21]. Both models are based on the assumed equilibrium between two conformational states of the enzyme; a relaxed (R) and tense (T) state. The main difference between these theories rests in how conformational transitions proceed upon binding of ligands. While the MWC model demands maintenance of conformational symmetry, the KNF model does not and instead requires strict induced fit. Although these models can be considered as special cases of more general models, these generalizations have so far not proven to be useful. In fact, the MWC model is regarded the model of choice for describing allosteric and cooperative interactions [22]. Moreover, it has been demonstrated that the MWC model can be cast in a convenient mathematical form [23], which can be combined with the elementary reaction formalism.

In the current work, we present a General Reaction Assembly and Sampling Platform (GRASP) capable of parameterizing and sampling the kinetics of any oligomeric enzyme by using minimal reference and biochemical mechanistic data. Parameterization combines the generalized MWC model for modelling the kinetics of oligomeric enzymes with the elementary reaction formalism for deriving thermodynamically consistent catalytic expressions. By employing a convenient normalization at the elementary reaction level [24], we are able to describe reaction kinetics provided a reference flux and the thermodynamic affinity of the reaction at the reference point. Using an accurate parameterization necessarily requires a large number of parameters. An advantage is that the parameterization retains all intrinsic thermodynamic constraints between kinetic parameters. We designed an efficient sampler producing thermodynamically consistent parameters obeying the principle of microscopic reversibility. Using an efficient Monte Carlo sampling technique that exploits the shape of the sampling space, we ensure high parameter quality and low parameter rejection rates. The framework is demonstrated through exploration of the full kinetic space of reactions, assessing the impact of thermodynamic affinity, reaction molecularity and mechanisms, as well as modelling complex kinetic behaviours.

## Models

### General framework for modelling metabolic reactions

In this article, we shall describe a general framework that enables parameterization and sampling of kinetic parameters consistent with thermodynamic constraints. The framework employs the MWC model, which provides the basis for modelling most cooperative and allosteric behaviours of multimeric enzymes [20]. The classical formulation including detailed assumptions and limitations are described in S1 Text. Our framework is based on the recasting of the model developed by Popova and Sel’kov [23,25–27], in which the velocity of reaction of any oligomeric enzyme is expressed as the product of *two independent functions*,

$$v=\Phi_{\text{catalytic}}\cdot \Psi_{\text{regulatory}}$$ (1)

where Φ_catalytic_ represents a rate law function for the protomers in the so-called relaxed (R) conformation, and Ψ_regulatory_ denotes a regulatory function describing the conformational mechanism of transition from a so-called tense (T) to the relaxed conformation. Equation 1 provides a general and simple interpretation of the kinetics of an oligomeric enzyme. Firstly, the shape of the catalytic function is determined *only* by the mechanism of elementary interactions between substrates and products with one active site of the enzyme (catalytic mechanism). Secondly, the regulatory function is *invariant* with respect to the action mechanism of the catalytic sites. Thus, if one has information about the conformational mechanism of the enzyme, *i*.*e*. number of subunits, transitions, allosteric sites and effectors, and possesses an expression representing the catalytic mechanism for the conversion of substrates to products, the catalytic and regulatory functions can be written as follows [23],

$$\Phi_{\text{catalytic}}=n\cdot v_{\text{R}}$$ (2)

$$\Psi_{\text{regulatory}}=\frac{1+\left(v_{\text{T}}/v_{\text{R}}\right)Q}{1+Q}$$ (3)

where *v*_R_ and *v*_T_ represent the velocity of reaction for the R and T conformations of the oligomeric enzyme, respectively (both states follow the same reaction mechanism as protomers are identical), and *Q* is a function that determines the current ratio between the R and T states (see later).

**Fig 1:**
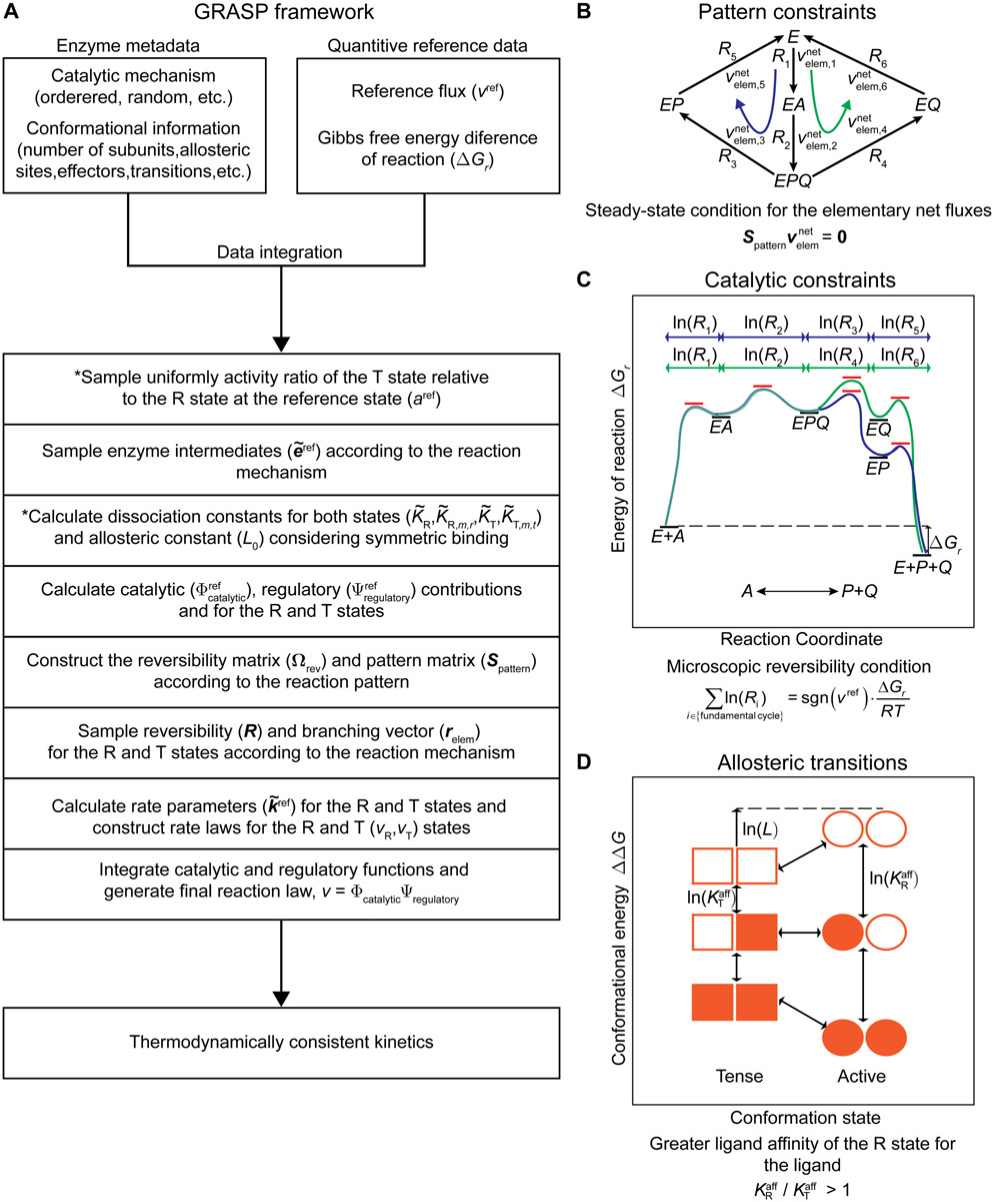
General framework for thermodynamically consistent parameterization and efficient sampling of metabolic reactions. (A) General Reaction Assembly and Sampling Platform (GRASP) workflow. The steps indicated by * are only required for parameterizing and sampling allosteric reactions. (B) Example of pattern constraints present in a random-order Uni-Bi mechanism with the formation of a ternary complex (*EPQ*). The intermediate *EPQ* splits to the *EP* and *EQ* enzyme intermediates. This behaviour can be modelled by uniformly sampling the solution space for the steady-state elementary net fluxes $v_{\text{elem}}^{\text{net}}$, which captures the stoichiometric properties of the reaction pattern. (C) Illustration of energetic constrains in the previous mechanism. There are 2 possible cycles converting *A* into *P+Q*, namely: *E* → *EA* → *EPQ* → *EP* → *E* and *E* → *EA* → *EPQ* → *EQ* → *E*. According to the principle of microscopic reversibility both pathways must be energetically equivalent as they execute the same reaction. Thermodynamic constraints for both paths are illustrated in the free energy graph. (D) Thermodynamic constraints on the equilibrium allosteric constant (*L*) within the generalized MWC model. The value of *L* depends on the ligand affinity of the active and tense states. Notably, in the absence of ligand the allosteric constant *L* favours the tense state, whereas with increasing concentrations of ligand the active state becomes more favoured (lower conformational energy).

The generalized MWC model enables parameterization of the kinetics of any oligomeric enzyme by decomposing the reaction velocity into two independent functions. An important feature of this parameterization is that it enables inclusion of fundamental thermodynamic relations between kinetic parameters, as it is compatible with the elementary reaction formalism. Some of these relations are lost when using other parameterizations. A complete overview of the proposed framework is depicted in Fig. 1A. In the following, we present a systematic method for parameterizing and sampling thermodynamically consistent kinetics.

Before considering complex cooperative or allosteric mechanisms, we will consider a simple non-allosteric enzyme, *i*.*e*. *n* = 1 and *L* = 0 (no tense state) or *L* = ∞ (no relaxed state), the resulting flux at any reference state is purely due to the catalytic function.

$$\begin{array}{l} \Psi_{\text{regulatory}}=1\\ v=\Phi_{\text{catalytic}}=v_{\text{R}} \end{array}$$ (4)

### Parameterization and sampling of the catalytic mechanism

Every enzymatic reaction can be broken into simple reversible steps called elementary reactions. Using the law of mass action, the rate of each elementary reaction is written as [2],

$$\begin{array}{l} v_{\text{elem}}=k\cdot x\cdot e\text{ for binding steps}\\ v_{\text{elem}}=k\cdot e\text{ for dissociation steps} \end{array}$$ (5)

where *k* is a microscopic rate constant, *x* is the reactant concentration and *e* is the concentration of enzyme intermediate involved in the elementary step. Typically, the absolute values of the metabolite and total enzyme concentrations are not known (although physiological ranges can be estimated). To overcome this limitation, normalization of all the variables around a reference point (steady-state flux) is a convenient strategy. Following the scaling procedure employed by Tran et al. [24], normalization of these variables yields,

$$\begin{array}{l} v_{\text{elem}}=\left(k\cdot e_{\text{total}}^{\text{ref}}\cdot x^{\text{ref}}\right)\cdot \left(\frac{x}{x^{\text{ref}}}\right)\cdot \left(\frac{e}{e_{\text{total}}^{\text{ref}}}\right)=\tilde{k}\cdot \tilde{x}\cdot \tilde{e}\text{ for binding steps}\\ v_{\text{elem}}=\left(k\cdot e_{\text{total}}^{\text{ref}}\right)\cdot \left(\frac{e}{e_{\text{total}}^{\text{ref}}}\right)=\tilde{k}\cdot \tilde{e}\text{ for dissociation steps} \end{array}$$ (6)

The normalized metabolite concentrations are unitary at the reference point $\left({\tilde{x}}^{\text{ref}}=1\right)$, which is used extensively in developing an efficient sampling strategy.

Rate laws can be derived from the enzyme mechanism and the microscopic rate constants using the King-Altman’s method [4]. In order to sample kinetics, we can in principle sample the rate constants directly. However, this leads to an inefficient sampler, where thermodynamic constraints can only be validated after sampling resulting in a high rejection rate. Instead, we design the sampling procedure to incorporate constraints directly without the need of assuming any particular distribution for the rate constants.

#### Sampling enzyme intermediate abundances

It can be shown that Equation 6 can be written in a simple generic form for the computation of the rate parameters at the reference state (S1 Text),

$$v_{\text{elem}}^{\text{ref}}=P({\tilde{e}}^{\text{ref}})\cdot \tilde{k}$$ (7)

where $v_{\text{elem}}^{\text{ref}}$ is the vector of elementary fluxes, $P({\tilde{e}}^{\text{ref}})$ is a diagonal matrix function of the enzyme intermediate abundances vector ${\tilde{e}}^{\text{ref}}$ and $\tilde{k}$ is a vector of rate constants. Notably, the enzyme intermediate abundances sums to one and can be readily sampled using appropriate probability distributions. Specifically, we seek to uniformly sample enzyme complex abundances subject to

$$\sum_{i=1}^{p}{\tilde{e}}_{i}=1$$ (8)

This problem can be viewed as uniformly sampling from the surface of a *p*-dimensional simplex, which is equivalent as sampling from a multivariate Dirichlet distribution with hyper-parameter vector **1** [28]. This distribution can be easily constructed and readily sampled using Gamma distributions [29].

#### Sampling reversibilities

The rate constants $\tilde{k}$ are subject to thermodynamic constraints, which can be exploited in sampling by introducing a reversibility parameter (*R*_*i*_) for each elementary reaction step describing the ratio of the reverse and forward elementary fluxes [24].

$$R_{i}={\left(\frac{v_{\text{elem,}i}^{\text{reverse}}}{v_{\text{elem,}i}^{\text{forward}}}\right)}^{sgn\left(v^{\text{ref}}\right)}$$ (9)

This parameter ranges between 0 and 1 with its particular value constrained by the values of the other reversibilities in the pattern according to the principle of microscopic reversibility. Briefly, this principle states that for any reaction at equilibrium the frequency of transitions in both directions is the same for each individual reaction step. The same applies for non-sequential reaction (random-order) mechanisms at equilibrium provided that the alternative paths are equivalent as far as kinetic order is concerned [30]. In the reference state considered here, the enzymes are not at equilibrium and the probability ratio of the forward and reversed trajectories is not unity (minimum entropy condition), but rather is given by the exponential of the total change in entropy along the forward process. We have extended the principle of microscopic reversibility to the non-equilibrium steady-steady flux (S1 Text) producing the following criterion,

$$\underset{i\in \text{ fundamental cycle}}{\sum \ln(R_{i})}=\mathrm{sgn}\left(v^{\text{ref}}\right)\cdot \frac{\Delta G_{r}}{RT}$$ (10)

where Δ*G*_*r*_ represents the Gibbs free energy difference of reaction, *R* denotes the universal gas constant, *T* is the absolute temperature and *v* ^ref^ denotes the reaction reference flux. In Equation 10 the sum is made over all sets of reversibilities capable of carrying out the reaction. Such sets are called fundamental cycles and it has been demonstrated that a necessary and sufficient condition for the system satisfying the microscopic reversibility principle, is for each of these cycles to satisfy the criterion (Fig. 1B-C) [31]. The fundamental cycles are found by traversing the edges of a graph connecting substrates to products and translating the paths into linear relations constraining different reversibility sets. A reversibility matrix (Ω_rev_) is constructed that contains all the thermodynamic constraints in the reversibility vector ln(***R***).

$$\Omega_{\text{rev}}\cdot \ln(R)=\mathrm{sgn}\left(v^{\text{ref}}\right)\cdot \frac{\Delta G_{r}}{RT}$$ (11)

In order to sample the reversibilities, they are first normalised so that they can be sampled from the unitary simplex,

$$\ln({\overset{⌢}{R}}_{i})=\frac{\ln(R_{i})}{\mathrm{sgn}\left(v^{\text{ref}}\right)\cdot \Delta G_{r}/RT}$$ (12)

where ${\overset{⌢}{R}}_{i}$ represents the scaled reversibility. Using this transformation, Equation 11 can be cast into a more convenient form.

$$\begin{array}{l} \Omega_{\text{rev}}\cdot \ln(\overset{⌢}{R})=1\\ 0\leq ln({\overset{⌢}{R}}_{i})\leq 1 \end{array}$$ (13)

For compulsory-order mechanisms, Ω_rev_ = **1** ^T^, and the condition in Equation 13 is the same as the enzyme intermediate abundances sampling case (Equation 8). In order to sample random-order mechanisms, the Dirichlet distribution can be used in conjunction with Linear Programming (LP) techniques as shown in Equation 14. For each scaled reversibility, the lower bound is randomly sampled using the Dirichlet distribution and later Equation 13 is solved by maximizing the sum of the reversibilities. By fixing the lower bound of each reversibility, we randomly generate points at different corners of the feasible hyperspace spanning all the solutions described by Equation 13.

$$\begin{array}{l} lb\sim Dirichlet(1)\\ \max1^{T}ln(\overset{⌢}{R})\\ \text{s.t.}\\ \Omega_{\text{rev}}\cdot \ln(\overset{⌢}{R})=1\\ lb\leq ln(\overset{⌢}{R})\leq 1 \end{array}$$ (14)

Using a sampled set of reversibilities satisfying Equation 11 and imposing the steady-state condition on each elementary reaction, *i.e. $v_{\text{elem,}i}^{\text{net}}=v_{\text{elem,}i}^{\text{forward}}-v_{\text{elem,}i}^{\text{reverse}}$*, the elementary flux vector can be computed as the product of two elements,

$$v_{\text{elem}}^{\text{ref}}=\Gamma \cdot r_{\text{elem}}$$ (15)

where **Γ** is a diagonal matrix function of the reversibilities and ***r***_elem_ is a branching vector taking into account the pattern stoichiometry (see below). The diagonal elements of **Γ** depend on the direction of the elementary reactions according to,

$$\begin{array}{l} \Gamma_{i,i}^{\text{forward}}:=\frac{1}{1-R_{i}^{sgn\left(v^{\text{ref}}\right)}}\\ \Gamma_{i,i}^{\text{reverse}}:=\frac{R_{i}^{sgn\left(v^{\text{ref}}\right)}}{1-R_{i}^{sgn\left(v^{\text{ref}}\right)}} \end{array}$$ (16)

Finally, the constant rate vector $\tilde{k}$ can be calculated by combining Equations 7 and 15 using the general equation.

$$\tilde{k}=P^{-1}({\tilde{e}}^{\text{ref}})\cdot \Gamma (R)\cdot r_{\text{elem}}$$ (17)

The matrix is invertible provided that $P({\tilde{e}}^{\text{ref}})$ is diagonal with only non-zero elements. For a complete derivation of this equation see the S1 Text. Calculation of the rate parameters as presented here ensures that they satisfy the fundamental thermodynamic principles under non-equilibrium conditions.

#### Sampling elementary flux vectors

In order to determine the branching vector, ***r***_elem_, one needs to take into account the stoichiometry of the reaction pattern. This can be achieved by formulating and solving the mass balances for the forward and reverse elementary reactions (Equation 18).

$$S_{\text{pattern}}\cdot \left(v_{\text{elem}}^{\text{forward}}-v_{\text{elem}}^{\text{reverse}}\right)=S_{\text{pattern}}\cdot v_{\text{elem}}^{\text{net}}=0$$ (18)

Essentially, all patterns can be considered cycles that transform substrates into products given that Δ*G*_*r*_<0 (Fig. 1B). The solution space is infinite as cycles are per definition indeterminate. However, the sampling space can be constrained by formulating two additional restrictions: (1) elementary net fluxes are non-negative and (2) the maximum elementary net flux in the pattern is equal to the reference flux for the R and T protomers (Equation 19).

$$\begin{array}{l} v_{\text{elem}}^{\text{net}}\geq 0\\ max\left(v_{\text{elem}}^{\text{net}}\right)=v^{\text{ref}} \end{array}$$ (19)

The set of particular solutions for $v_{\text{elem}}^{\text{net}}$ that satisfy both Equations 18 and 19 will be called ***r***_elem_. Direct sampling $v_{\text{elem}}^{\text{net}}$ from this subspace can be difficult. An alternative approach is to express all the paths solving Equation 18 as a linear combination of the null space basis of ***S***_pattern_ (***N***_pattern_), sample uniformly the weights (***w***) that yield a non-negative solution and then normalize (Fig. 1B). This procedure is summarized in Equations 20 and computationally produces valid branching vectors more efficiently than direct sampling.

$$\begin{array}{l} v_{\text{elem}}^{\text{net}}=N_{\text{pattern}}\cdot w\\ r_{\text{elem}}=v^{\text{ref}}\cdot \frac{v_{\text{elem}}^{\text{net}}}{max\left(v_{\text{elem}}^{\text{net}}\right)} \end{array}$$ (20)

In summary, for a monomeric enzyme we use the elementary reaction formalism and sample the microscopic rate constants indirectly by sampling the three elements in Equation 17: the reference state values of the enzyme intermediates, the reversibilities and the branching vector. This sampling strategy ensures that any parameter set sampled satisfies both pattern and kinetic constraints (Fig. 1B-C).

### Sampling functional contributions: catalytic and regulatory effects

Returning to the generalised MWC model, Equations 1–3, we note that the catalytic and regulatory contributions are confounded in the case of allosteric enzymes. Even if the reaction flux is known in a particular state, the particular values of both functions are unknown. To resolve this, we need to elucidate the contributions of the relaxed and tense conformations. The regulatory function is always less than or equal to 1, as the catalytic activity of the T state is less than the R state [32], and we introduce the activity ratio (*a* ^ref^) of a tense protomer at the reference state and sample this uniformly.

$$a^{\text{ref}}=\frac{v_{\text{T}}^{\text{ref}}}{v_{\text{R}}^{\text{ref}}}\sim \text{Uniform}(0,1)$$ (21)

Given *a* ^ref^ we can rearrange Equations 2 and 3 to calculate the contributions of each state

$$\begin{array}{l} v_{\text{R}}^{\text{ref}}=\frac{\Phi_{\text{catalytic}}^{\text{ref}}}{n}=\frac{v^{\text{ref}}}{n\Psi_{\text{regulatory}}^{\text{ref}}}\\ v_{\text{T}}^{\text{ref}}=a^{\text{ref}}\cdot v_{\text{R}}^{\text{ref}} \end{array}$$ (22)

The catalytic mechanism is assumed identical for the R and T state and we sample this mechanism twice using the two different reference points to generate feasible parameterisations for the R and T state.

The final step is to generate a sample of parameters for the *Q* function that satisfy Equation 3 in the reference point. The *Q* function can be expressed as [33],

$$Q=L_{0}\cdot {\left(\frac{{\tilde{e}}_{\text{R0}}(x,k_{\text{R}})}{{\tilde{e}}_{\text{T0}}(x,k_{\text{T}})}\cdot \frac{\prod_{i=1}^{m}\sum_{j=1}^{r}\left(1+x_{\text{F},i,j}/K_{\text{T,}i,j}\right)}{\prod_{i=1}^{m}\sum_{j=1}^{t}\left(1+x_{\text{F},i,j}/K_{\text{R},i,j}\right)}\right)}^{n}$$ (23)

where *L*_0_ is the allosteric constant between the R and T states in the absence of ligands, *x*_F,*i*,*j*_ represent effector concentrations binding to specific allosteric sites, *K*_R,*i*,*j*_ and *K*_T,*i*,*j*_ denote the effectors dissociation constants for each state, ${\tilde{e}}_{\text{R0}}$ and ${\tilde{e}}_{\text{T0}}$ are the free enzyme fractions in both conformational states as function of the respective rate parameters (***k***_R_,***k***_T_) and reactant concentrations (***x***), *m* represents the number of allosteric sites, and finally, *r* and *t* are the number of positive and negative effectors binding to the allosteric sites in the R and T states, respectively. Here we have assumed that the allosteric activators and inhibitors only bind to the R and T, respectively [32], although this constraint can be relaxed.

In the reference point, *Q* does not depend on the reactant and effector concentrations as they are defined as unitary at the reference point. We can determine all parameters in the *Q* function based on sampled enzyme abundances (refer to Equation 8).

In the case of the conformational transitions, the change of the Gibbs free energy of conformations between the R and T states is constrained by the ligand affinity of the two states. These transitions point to a higher relative abundance of the free enzyme in the T state in the absence of substrate, whereas in the presence of substrate the R state is more favoured [32]. Ultimately, the latter yields *L*>1 and a ratio of affinity constants for both states $K_{\text{R}}^{\text{aff}}/K_{\text{T}}^{\text{aff}}>1$ or equivalently *K*_T_/*K*_R_ > 1 in the case of dissociation constants (Fig. 1D).

Colosimo et al. [34] have derived a simple expression to determine the allosteric constant in the absence of ligands assuming symmetric binding for the two states (Equation 24).

$$L_{0}={\left(\frac{K_{\text{T}}}{K_{\text{R}}}\right)}^{n/2}$$ (24)

To estimate the dissociation constants in Equation 24, we can make use of the equilibrium assumption between the R and T states and the unitary ligand concentration normalized at the reference state.

$$L_{0}={\left(\frac{{\tilde{e}}_{\text{T0}}^{\text{ref}}/{\tilde{e}}_{\text{T}1}^{\text{ref}}}{{\tilde{e}}_{\text{R0}}^{\text{ref}}/{\tilde{e}}_{\text{R1}}^{\text{ref}}}\right)}^{n/2}$$ (25)

In Equation 25, $\left({\tilde{e}}_{\text{0}}^{\text{ref}}\right)$ and $\left({\tilde{e}}_{1}^{\text{ref}}\right)$ denote the enzyme fractions free and bound to the ligand for the R and T states at the reference state. The same principle can be used to calculate the effectors dissociation constants. Calculation of each constant is achieved using the following general formula for the allosteric site *m*, and effector *r* binding to the R state and effector *t* binding to the T state.

$$\begin{array}{l} {\tilde{K}}_{\text{R},m,r}=\frac{e_{\text{R0}}^{\text{ref}}/e_{\text{total}}\cdot x_{\text{F},m,r}^{\text{ref}}}{e_{\text{R}1}^{\text{ref}}/e_{\text{total}}}=\frac{{\tilde{e}}_{\text{R}0}^{\text{ref}}}{{\tilde{e}}_{\text{R}1}^{\text{ref}}}\cdot x_{\text{F},m,r}^{\text{ref}}\\ {\tilde{K}}_{\text{T},m,t}=\frac{e_{\text{T0}}^{\text{ref}}/e_{\text{total}}\cdot x_{\text{F,}m,t}^{\text{ref}}}{e_{\text{T}1}^{\text{ref}}/e_{\text{total}}}=\frac{{\tilde{e}}_{\text{T0}}^{\text{ref}}}{{\tilde{e}}_{\text{T1}}^{\text{ref}}}\cdot x_{\text{F,}m,t}^{\text{ref}} \end{array}$$ (26)

In particular, the absolute concentrations of the allosteric effectors $\left(x_{m,r}^{\text{ref}},x_{m,t}^{\text{ref}}\right)$ need not to be known, as they are scaling factors for the absolute effector concentrations in Equation 23 which are unitary at the reference state.

### Parameter set accuracy check

The sampling procedure generates only feasible parameter sets. Since this parameterization is built upon a reference point, this can be validated by confirming that the parameter set produces the reference flux at the reference point, *i*.*e*. when the normalized metabolite concentrations are unitary. We control the numerical accuracy of parameter sets by accepting only sets achieving the reference flux within a tolerance, *e.g. ε* = 10^-8^,

$$\left|\Phi_{\text{catalytic}}^{\text{ref}}\cdot \Psi_{\text{regulatory}}^{\text{ref}}-v^{\text{ref}}\right|<\varepsilon$$ (27)

In general, rejected instances are insignificant and normally represent less than 0.01% of the sampled models.

If the reference values for the total enzyme and metabolite concentrations are known, they can be used to transform the set of scaled rate constant into absolute constants. Microscopic rate constants are commonly difficult to measure; therefore standard macroscopic constants are preferred to parameterize rate expressions. Transformation of rate constants into macroscopic rate constants can be performed following Cleland’s rules [6], which are consistent with the Haldane relationships. In this manner, estimated macroscopic constants can readily be compared with available experimental data.

### Elasticity analysis of the velocity of reaction

In order to determine the impact of reactant perturbations on the reaction rate, we estimated the reaction elasticities upon an infinitesimal variation in the concentration of substrates and products [2]. The partial derivatives were calculated using a central difference approximation,

$$\varepsilon_{\tilde{x}}^{v}=\frac{\tilde{x}}{v}{\frac{\partial v}{\partial \tilde{x}}|}_{\left(v^{\text{ref}},{\tilde{x}}^{\text{ref}},\tilde{k}\right)}\approx \frac{{\tilde{x}}^{\text{ref}}}{v^{\text{ref}}}\cdot \frac{v\left({\tilde{x}}^{\text{ref}}+\Delta h,\tilde{k}\right)-v\left({\tilde{x}}^{\text{ref}}-\Delta h,\tilde{k}\right)}{2\Delta h}$$ (28)

where ${\tilde{x}}^{\text{ref}}$ represents the perturbed normalized reactant concentration, $\tilde{k}$ denotes the rate constants vector and Δ*h* is the size of the perturbation. Given that the perturbations for reactant concentrations are performed in the vicinity of the reference state, *i.e. ${\tilde{x}}^{\text{ref}}=1$*, a uniform step size of 10^-2^ equivalent to a 1% change was employed for all calculations.

### Computational implementation

The implementation and execution of this workflow was performed in MATLAB 2013a (The MathWorks, Natick, MA). Definition of the reversibility matrix was achieved using appropriate MATLAB functions from the Bioinformatics toolbox. Automated derivation of rate laws was achieved using King-Altman’s method for finding valid reaction patterns [4]. Qi et al. [35] have recently reported an efficient algorithm (*KAPattern*) employing topological theory of linear graphs for accomplishing this goal. By employing this algorithm, we derived the enzyme intermediates abundance functions which we then assembled to build the final velocity rate. All computations were run on a Dell OptiPlex 990 Desktop (Intel Core i5-2400, 4 GB ram, Microsoft Windows 7, x86-based architecture).

## Results

We have developed an efficient algorithm for parameterizing and sampling a very broad family of enzyme mechanisms (Fig. 1). In the following, we will describe several applications of this platform to assess the impact of thermodynamic affinity, reaction molecularity and mechanisms, as well as modelling complex kinetic behaviours.

### Dissecting enzyme catalysis: assessing the impact of reaction molecularity

The connection between reaction thermodynamics and kinetics can be normally found in the form of the Haldane relationships [5]. Depending on the mechanism of reaction, these relations relate the values of the macroscopic kinetic constants, *i*.*e*. dissociation and catalytic constants, with the apparent equilibrium constant of the reaction. It would be interesting to derive similar relations under non-equilibrium steady-state conditions, provided that biological systems operate in this regime. It has been demonstrated that the analysis of the relation between the thermodynamic affinity (represented by Δ*G*_*r*_/*RT*) and the reaction velocity can still be performed as if it were at equilibrium, by displacing the reference point from the equilibrium to another appropriate reference state [36]. As such, relations analogous to the Haldane relationships can be derived at this reference point (S1 Text). For example, for a Uni-Uni reaction converting a substrate *A* into a product *P* the following relation can be derived,

$$\exp\left(\frac{\Delta G_{r}}{RT}\right)=\frac{{\tilde{k}}_{-1}{\tilde{k}}_{-2}{\tilde{k}}_{-3}}{{\tilde{k}}_{1}{\tilde{k}}_{2}{\tilde{k}}_{3}}=\frac{{\tilde{k}}_{\text{cat,-}}\cdot {\tilde{K}}_{A}}{{\tilde{k}}_{\text{cat,+}}\cdot {\tilde{K}}_{P}}$$ (29)

where ${\tilde{k}}_{i}$ denote the rate constants at the reference state, ${\tilde{K}}_{A}$ and ${\tilde{K}}_{P}$ represent the normalized dissociation constants for *A* and *P* at the reference state, respectively, and ${\tilde{k}}_{\text{cat,+}}$ and ${\tilde{k}}_{\text{cat,-}}$ are the scaled catalytic constants for the forward and reverse reactions. It can be demonstrated that the following generalized equation holds true for any reaction mechanism,

$$\frac{\Delta G_{r}}{RT}=\underset{\text{catalytic}}{\ln\left(\frac{{\tilde{k}}_{\text{cat,-}}}{{\tilde{k}}_{\text{cat,+}}}\right)}+\underset{\text{binding}}{\sum_{i}^{s}\sum_{j}^{p}\ln\left(\frac{{\tilde{K}}_{i}}{{\tilde{K}}_{j}}\right)}$$ (30)

where *s* and *p* denote the number of substrates and products, respectively. We have designated the first term on the right-hand side of Equation 30 the catalytic (turnover) term, while the second is called the binding (saturation) term. Notably, the catalytic term is independent of the reactant concentrations at the reference state, as opposed to the saturation term. In fact, it can be shown that ${\tilde{K}}_{A}=K_{A}/A^{\text{ref}}$, which can be regarded as the degree of saturation of the enzyme for reactant *A*. In this way, Equation 30 enables the energetic analysis of any reaction by decomposing it in two contributions: (1) catalysis (how efficient is the enzyme converting substrates into products at the reference state) and (2) binding (how saturated is the enzyme at the reference state). More importantly, this relation imposes a natural trade-off in enzyme catalysis. Greater contributions of the catalytic term suggest a higher enzymatic efficiency in the conversion of substrates to products, but a suboptimal saturation of the enzyme, *i.e. K_A_/A* ^ref^ high. On the contrary, high saturation contributions suggest lower conversion efficiency of substrates into products, *i*.*e*. ${\tilde{k}}_{\text{cat,-}}/{\tilde{k}}_{\text{cat,+}}$ is relatively higher compared to the binding term.

**Fig 2:**
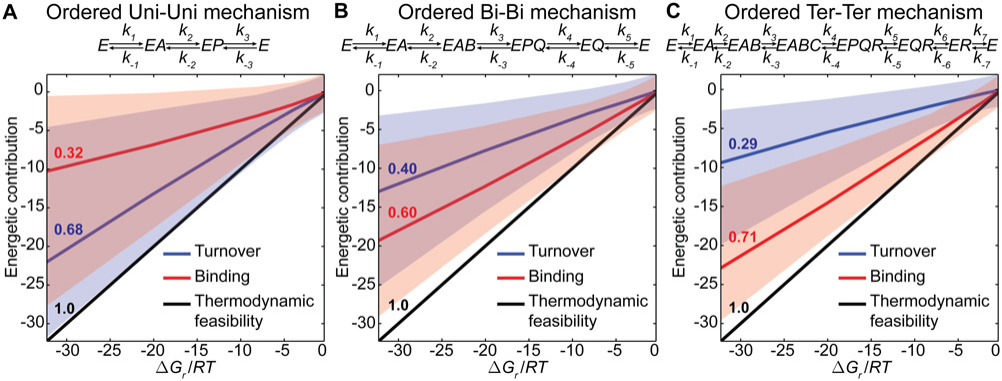
Energetic landscape of the contributions of the catalytic and binding terms for ordered reaction mechanisms with different molecularities. Molecularities analysed include (A) Uni-Uni, (B) Bi-Bi and (C) Ter-Ter reaction mechanisms. In each case, the following Gibbs free energy differences were considered: -1, -5, -10, -20, -30, -50 and -80 kJ/mol and a reference flux of 1 mM/min was assumed. For this analysis, 10^4^ parameter sets were sampled. The blue and red lines denote the median of the distributions for the catalytic and binding terms of the reaction energetics, respectively, while the shaded areas represent the 95% confidence regions for the respective contributions. The black line represents the sum of the two contributions which yields Δ*G*_*r*_/*RT*. As it can be observed, both contributions decrease linearly with decreasing Δ*G*_*r*_/*RT*. In addition, the higher the molecularity the higher the relative importance of the binding term. The numbers showed above each line denote their slope and quantify the extent of their energetic contributions. Notably, the sum of all contributions must be one.

To explore the consequences of this trade-off in enzymatic reactions, we uniformly sampled the kinetic space of three ordered mechanisms following different molecularities: Uni-Uni, Bi-Bi and Ter-Ter, under different Gibbs free energy differences and a constant reference flux. S1 Table shows the definition of the dissociation and catalytic constants in terms of the rate constants using Cleland’s definitions. We performed the analysis considering conditions close to equilibrium (-1 kJ/mol) to far from equilibrium (-80 kJ/mol) (Fig. 2). Notably, the average conversion and saturation contributions ratio remains constant for different Δ*G*_*r*_/*RT* for all the kinetics sampled. As expected, the higher the molecularity of the reaction, the higher the contribution of the binding term. The Uni-Uni mechanism exhibits a low average binding contribution (32%), which increases for the Bi-Bi mechanism (60%) and further for the Ter-Ter mechanism (71%). These results suggest that catalysis in multi-substrate enzymes is a process strongly driven by the degree of saturation of the enzyme and to a lesser extent by the actual conversion of substrates into products.

Uniform sampling of the kinetic space enables an unbiased appraisal of the relation between thermodynamics and kinetics. For the aforementioned cases, the sums of the blue and red areas represent the 95% confidence region of all the thermodynamically feasible parameter sets. Interestingly, for all the tested mechanism, the feasible area increases with higher driving force (Δ*G*_*r*_/*RT* more negative). Such increased area point to a greater diversity of feasible parameter sets under more thermodynamically favourable conditions. On the contrary, more homogeneous parameter sets can be found closer to equilibrium, *i*.*e*. different parameter sets have similar energetic contributions. The latter suggests that sampled parameter sets might have similar kinetic behaviours closer to equilibrium. The next analysis will provide additional support for this assertion.

### Revealing the impact of thermodynamics on enzyme kinetics

**Fig 3:**
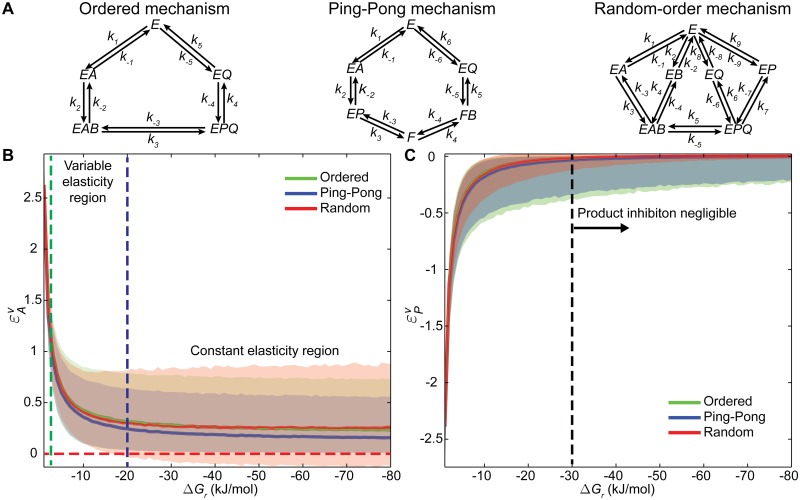
Revealing the impact of thermodynamics on enzyme kinetics. (A) Schematic representation of the bimolecular mechanisms considered for the analysis. (B) Substrate elasticities for three mechanisms considered: ordered (green), ping-pong (blue) and random-order (red) at different Gibbs free energy differences of reaction ranging from -1 to -80 kJ/mol. Elasticities were calculated every -1 kJ/mol interval by sampling 10^4^ instances each time. In this panel, each line represents the median of the respective elasticity distribution, while the shaded areas denote the 95% confidence regions. Depending on the chosen thermodynamic reference state, the elasticities can be almost constant for regions far from equilibrium (Δ*G*_*r*_ <-20 kJ/mol) or highly variable close to equilibrium (dashed blue line). The dashed green line represents the limit for the lineal regime of substrate elasticity variation, while the dashed red line denotes the zero limit. (C) Product elasticities for the same representative bimolecular mechanisms. The lines and shaded areas represent the same as for the substrate elasticities. Product inhibition is on average negligible for favourable thermodynamic conditions, *i*.*e*. Δ*G*_*r*_ < -30 kJ/mol (right side of the dashed blue line).

In the previous section, we assessed the effect of the reaction molecularity on enzyme catalysis. We next analysed the impact of thermodynamics on enzyme kinetics. To that end, we uniformly sampled the kinetic space of reactions with same molecularity. Since the majority of enzymes found in nature catalyse bimolecular reactions [2], we focussed our attention on the most representative bimolecular mechanisms. There are two general mechanisms for bimolecular reactions, the ping-pong mechanism in which a product is released before both substrates have reacted with the enzyme, and the ternary complex (sequential) mechanism in which the enzyme combines with both substrates before products are formed [6]. Sequential mechanisms can be further divided into random and ordered mechanisms. In random-order mechanisms, reactants can bind in either order, while in the ordered type sequential process one reactant always binds to a certain site before a second reactant binds to the other site [37]. The representation of all three mechanisms is shown in Fig. 3A. The impact of thermodynamics was evaluated in each case by calculating the reaction sensitivities for substrates and products, *i*.*e*. substrate and product elasticities, at different Gibbs free energy differences ranging from -1 (close to equilibrium) to -80 kJ/mol (practically irreversible). The 95% confidence regions for the calculated reaction elasticities for the above mentioned reaction mechanisms are shown in Fig. 3B-C.

The substrate and product elasticities strongly depend on the chosen thermodynamic reference state. Within the range of Δ*G*_*r*_ analysed, two reaction elasticity regions can be distinguished: a variable elasticity region (0>Δ*G*_*r*_>-20 kJ/mol) and a constant elasticity region (Δ*G*_*r*_<-20 kJ/mol). The former region can be further subdivided in two: a linear regime with a steep slope (0>Δ*G*_*r*_ >-2 kJ/mol) and a transition regime (-2>Δ*G*_*r*_>-20 kJ/mol). Notably, previous works have demonstrated an almost perfect linear correspondence between the thermodynamic affinity and the reaction flux close to equilibrium, *i*.*e*. 0>Δ*G*_*r*_ > -1.5 kJ/mol [38,39]. Such relationship has been shown to hold for Δ*G*_*r*_>-7 kJ/mol with less than 15% error [40]. Our simulation results are thus in line with what would be expected to be the kinetic behaviour close to equilibrium.

A remarkable feature of our sampling strategy is that it not only enabled analysis of the reaction elasticities behaviours close to equilibrium but also far from it. In the case of the reaction elasticities close to equilibrium, our sampling results show the substrate and product elasticities are respectively monotonically decreasing and increasing, reaching their respective maximum and minimum at equilibrium (Fig. 3B). This behaviour is consistent with previous analyses on the behaviour of the reaction elasticities in this region [2] and supports our intuition: reactions operating close to equilibrium are more susceptible to slight changes in the reactant concentrations, exhibiting greater changes upon these perturbations. On the other hand, our analysis far from equilibrium revealed both substrate and product elasticities reach a plateau at highly negative thermodynamic affinities for all the reaction mechanisms analysed. In the case of the substrate elasticity, this plateau stabilizes close to zero for highly negative Δ*G*_*r*_. Notably, the average substrate elasticity for thermodynamically favoured reactions is approximately 0.22 and not 0 as it would be expected. The latter is a direct result of our sampling strategy, which seeks to uniformly sample all the possible parameter sets that are consistent with the Haldane relationships at a chosen reference point. On the contrary, in the case of the product elasticity, the average elasticity consistently approaches zero for all the mechanisms analysed under favoured conditions. Furthermore, our sampling results suggest that product inhibition is almost negligible for thermodynamically favoured reactions, *i*.*e*. Δ*G*_*r*_<-30 kJ/mol (Fig. 3C). S1 Text provides an illustrative explanation of the asymptotic behaviour of the reaction elasticities near and far from equilibrium. Additionally, it is also important to highlight that the allowable elasticity regions becomes tighter as we move closer to equilibrium (Fig. 3B-C). The latter supports our earlier findings of sampling more homogenous parameter sets close to equilibrium. Indeed, the closer to equilibrium the tighter the feasible parameter space becomes, and thus, a more similar response upon reactant perturbations is obtained.

Finally, reaction mechanisms have similar elasticity behaviours across the analysed Δ*G*_*r*_ range. However, the average response of ordered and random mechanisms is more similar than the response of the ping-pong mechanism. This is most readily appreciated when comparing the substrate elasticities (Fig. 3B). As previously mentioned, the ping-pong mechanism executes a fundamentally different mechanism in which the release of the first product takes place before binding of the second substrate. The latter explains the slightly lower average substrate elasticity. Interestingly, negative substrate elasticities can be encountered in the random order mechanism for high thermodynamic affinities (approx. Δ*G*_*r*_<-18 kJ/mol), *i*.*e*. the addition of substrate decreases the velocity of reaction (elasticities below the red dashed line in Fig. 3B). This behaviour has been demonstrated previously for random-order mechanisms [41]. Theoretical analysis of these mechanisms has shown that they can give rise to apparent substrate inhibition or substrate activation [2]. For example, depending on the kinetic parameter values and the substrate concentrations, the reaction rate for the enzymes following this mechanism can display an apparent cooperative behaviour (sigmoidal reaction rate) upon addition of one substrate maintaining the other constant, while in the opposite situation they can exhibit substrate inhibition (the reaction rate pass through a maximum) [41]. Although this behaviour is not very common, there is evidence of such in the literature [42]. More importantly, our framework enabled unbiased sampling of all feasible kinetic behaviours, thereby revealing the impact of the thermodynamic affinity on the reaction rate.

### Sampling a rare kinetic event: Monomeric cooperativity of mammalian glucokinase

ATP-mediated phosphorylation of glucose represents the first step of the glycolysis. In mammals, this step is performed by four different isozymes (EC 2.7.1.1) located in different tissues. Hexokinases I-III are mainly located in the brain, muscle and erythrocytes [43], while hexokinase IV (glucokinase) is primarily located in the liver and pancreatic β-cells [44]. In the latter tissues, glucose phosphorylation is the rate-limiting step of glucose metabolism, and more importantly, it is ultimately responsible for the release of insulin into the bloodstream which maintains glucose homeostasis in the body.

**Fig 4:**
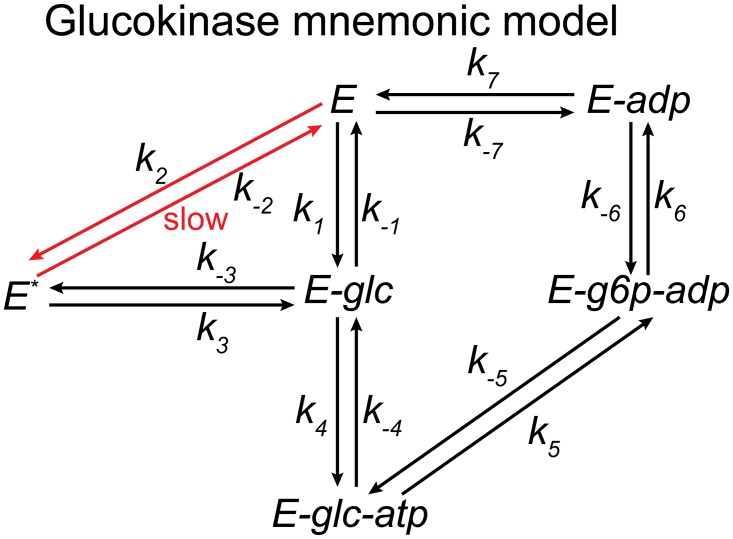
Schematic representation of the mnemonic model for the mammalian glucokinase. This model proposes a slow conformational transition from a low-affinity state (*E* ^*^) to a high-affinity state (*E*) that can be enhanced with increasing glucose concentration yielding the observed cooperative behaviour.

Unlike hexokinases I-III, glucokinase has unique kinetic features that enable its regulatory function. It displays a sigmoidal response upon increasing glucose concentration, *i*.*e*. positive cooperativity, but has a hyperbolic behaviour upon increasing MgATP^2-^, *i*.*e*. Michaelis-Menten kinetics [45]. This positive cooperativity for glucose is intriguing as the enzyme is monomeric, which contradicts standard cooperativity models and thus requires a conceptually different explanation. One of the simplest models capable of explaining this behaviour is the mnemonic model [46]. Briefly, this model proposes that the conformation of an enzyme following product release could be different from the initial enzyme state, *i*.*e*. the enzyme has memory. In the case of glucokinase, this model suggests a slow conformational transition from a low-affinity state (E*) to a high-affinity state (*E*), which ultimately carries out both catalysis and product release (Fig. 4). This transition can be enhanced with increasing glucose concentrations, yielding the observed positive cooperative behaviour on this substrate (kinetic cooperativity). Other possible explanations like a reaction mechanism with random addition of substrates has been discarded, as isotope exchange studies have demonstrated an ordered mechanism with glucose binding first [47].

One question that can be addressed using the current framework is how likely it is to encounter a particular kinetic behaviour. In this case, we are interested in estimating the frequency of positive cooperativity for glucose of the glucokinase. To this end, we uniformly sampled the kinetic space for this enzyme and counted the frequency of parameter sets displaying positive cooperativity for glucose (estimated Hill coefficient, *n*_H_>1). In order to mimic enzymatic assays conditions, *i*.*e*. high substrate concentrations and almost no products, a reference Gibbs free energy difference of -100 kJ/mol was used. Very similar results were found using down to -50 kJ/mol of Δ*G*_*r*_. The latter was expected as reaction elasticities were found to reach a plateau for Δ*G*_*r*_<-30 kJ/mol (Fig. 3B-C). Nevertheless, in order to ensure initial velocity conditions, we chose the former difference. The reference steady-state flux under this condition was set to the experimental value of 0.064 mM/min found in the literature [48].

**Fig 5:**
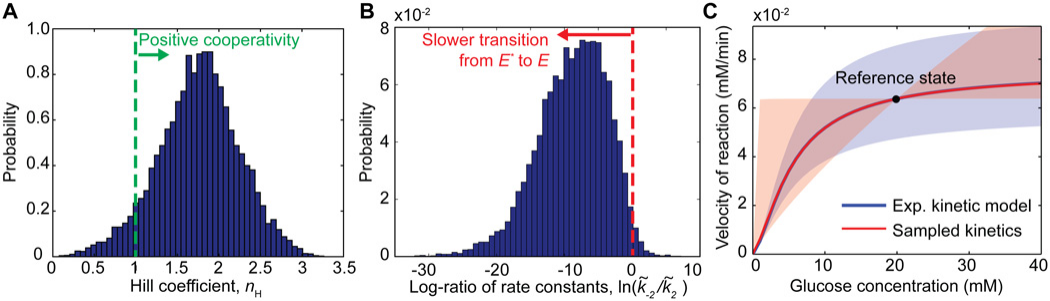
Sampling monomeric cooperativity of mammalian glucokinase. (A) Probability distribution of the Hill coefficient for the glucokinase. Approximately 93% out of 10^4^ sampled kinetics displays cooperative behaviour. (B) Probability distribution for the ratio of the forward and reverse rate constants from the low- to the high-affinity enzyme state. The great majority (98.1%) of the models exhibiting positive cooperativity agrees with a slow transition from the low- to the high-affinity enzyme states. (C) Comparison of the kinetic space described by the model developed by Storer and Cornish-Bowden [48] (blue) and the sampled kinetics using the mnemonic model (red). Each line represents the real kinetic behaviour described by the two models, while the shaded areas denote one-standard-deviation confidence regions for each approach.

Approximately 93% of the sampled kinetics displays positive cooperativity for glucose (Fig. 5A). However, a small portion of the models (~7%) displayed an apparent negative cooperative behaviour. The latter is possible due to the existence of competing parallel pathways in the reaction mechanism, *i*.*e*. *E* → *E-glc* → *E-glc-atp* → *E-g6p-adp* → *E-adp* → *E* and *E* → E* → *E-glc* → *E-glc-atp* → *E-g6p-adp* → *E-adp* → *E* (Fig. 4). As previously mentioned, reaction mechanisms with branched steps can exhibit apparent substrate inhibition depending on the kinetic parameter values and the substrate concentrations. In this case, this will depend on the value of the branching factor for the *E* → *E-glc* and *E* → E* steps. In particular, if a large elementary net flux is sampled for the *E* → E* step at the reference state, then the successive addition of glucose will inhibit the velocity of the reaction as opposed to enhancing it.

The great majority of the models displaying cooperativity for glucose are consistent with a slow transition from the low to the high-affinity enzyme state (98.1%) (Fig. 5B). This kinetic behaviour has been extensively studied and there is abundant supporting evidence [49]. Remarkably, the sampled kinetics contained the experimentally observed Hill coefficient for this enzyme (*n*_H,real_ = 1.70 ±0.1 [49]), within one standard deviation (*n*_H,sampled_ = 1.77±0.5), which confirms the suitability of the mnemonic model for modelling this kinetic behaviour. Fig. 5C shows a comparison of the fitted kinetic model by Storer and Cornish-Bowden [48] and the sampled kinetics using our framework. Better agreement between the models is encountered at high glucose concentrations (>20 mM), *i*.*e*. where the cooperative behaviour is less pronounced, while at lower glucose concentrations (<4 mM) slightly higher discrepancies can be observed. As expected, a perfect match between both models is found at the reference state, as by construction the proposed framework builds the parameterization at this point (*v* ^ref^ = 0.064 mM/min). Interestingly, the variability of the sampled kinetics is not proportional to the glucose concentration as opposed to fitted model (Fig. 5C). For some glucose concentration regions, the kinetic behaviour of the sampled models displays a relative greater variability for low glucose concentrations (<20 mM) and a relative lower variability for higher concentrations. The latter shows that thermodynamically consistent parameter sampling can provide means for effectively accounting for the feasible kinetic space without the need for excessive data. Moreover, the addition of extra data will further reduce the feasible kinetic space for this particular mechanism. Taken together, these results show that by using this framework, exploration of the kinetic space for complex reactions can be achieved provided that the reaction mechanism is known and by setting the right thermodynamic constraints.

### Allosteric regulation: Modelling co-activation of *Escherichia coli* PEP carboxylase (PEPC)

**Fig 6:**
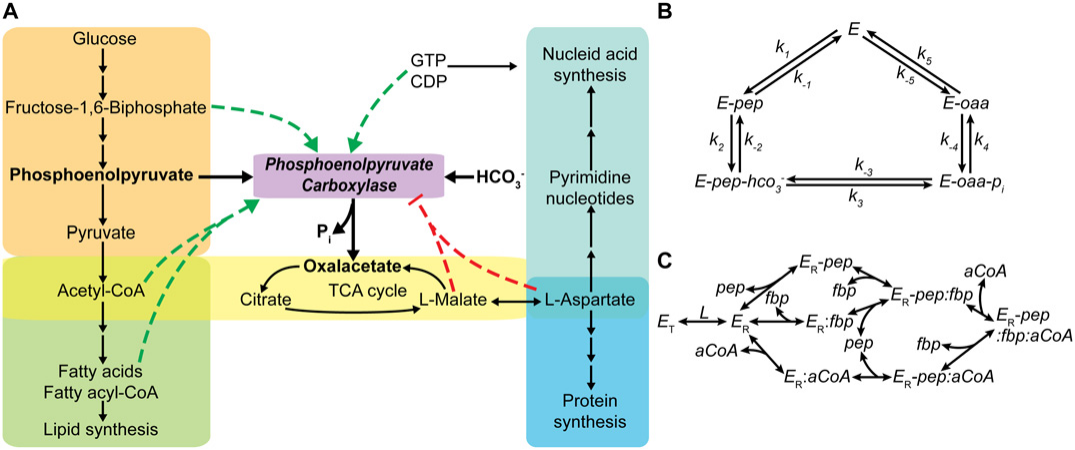
Complex allosteric regulatory interactions control the kinetic behaviour of phosphoenolpyruvate carboxylase (PEPC) in *E*. *coli*. (A) PEPC regulates anaplerotic metabolism in *E*. *coli* through several allosteric interactions. This enzyme carries out the conversion of phosphoenolpyruvate and bicarbonate into oxaloacetate and inorganic phosphate. All reactants are highlighted in black. The red and green dashed lines denote the inhibition and activation exerted by different effectors, respectively. (B) Ordered Bi-Bi mechanism for PEPC catalysis. Phosphoenolpyruvate, bicarbonate, oxaloacetate and orthophosphate are denoted by the abbreviations *pep*, *hco*_*3*_^*−*^, *oaa* and *p*_*i*_, respectively. (C) Mechanism of synergistic activation of PEPC transitions proposed by Smith et al. [57]. This mechanism considers two separate binding sites for the two types of activators (allosteric sites) and another for the substrates (catalytic site), each of them being capable of independently interacting with different enzymatic complexes. Notably, this model assumes the existence of the relaxed enzyme (active) only in the presence of activators, one of which may be the substrate *pep*.

Phosphoenolpyruvate carboxylase (EC 4.1.1.31) is one of the CO_2_-fixing enzymes present in the carbon central metabolism of many photosynthetic organisms as well as most non-photosynthetic bacteria and protozoa [50]. In glucose-limited *Escherichia coli* cultures, PEPC is involved in the only active anaplerotic reaction replenishing pools of intermediary metabolites of the tricarboxylic acid (TCA) cycle [51] (Fig. 6A). This enzyme catalyses the conversion of phosphoenolpyruvate (PEP) and bicarbonate into oxaloacetate and orthophosphate in the presence of Mg^2+^. This reaction is highly exergonic ($\Delta G_{r}^{o}$ = -43.2kJ/mol[52]), making it practically irreversible under physiological conditions. The catalytic mechanism of this enzyme has been elucidated with an ordered binding of phosphoenolpyruvate first and releasing of oxaloacetate as the final step (Fig. 6B) [50]. The regulatory mechanism behind the operation of this enzyme is much more complex. The enzyme is allosterically activated by acetyl-CoA, long-chain fatty acids and their acyl coenzyme A derivatives, fructose 1,6-bisphosphate (FBP) and the nucleotides guanosine-5’-triphosphate and cytidine-5’-diphosphate, whereas it is inhibited by L-aspartate and L-malate [53–56] (Fig. 6A). In particular, the mechanism of activation of this enzyme upon the combined action of FBP and acetyl-CoA has been studied in detail. These two activators bind to different allosteric sites, synergistically activating the tetrameric active form [57]. Alone FBP has been shown to exert little activation of the enzyme upon binding of PEP; however acetyl-CoA alone can greatly enhance the activation induced by FBP [58]. A plausible synergistic model capable of explaining this behaviour has been proposed by Smith et al. [57] and it is shown in Fig. 6C. Notably, this model is a hybrid between that of Monod et al. [20] and that of Koshland et al. [21] in that the mechanism of transitions requires the presence of the activators and/or ligand (PEP) for the enzyme to be active (induced fit), however it also assumes isomerization of all subunits to describe cooperative interactions (concerted model).

The particular kinetic features of the PEPC have been shown lately to play a key role in the regulation of anapleurosis in *E*. *coli*. Xu et al. [58] have demonstrated that *E*. *coli* is able to turn off PEP consumption quickly upon glucose removal thanks to the ultrasensitive response of the PEPC upon FBP depletion. Following glucose removal, depletion of FBP from 15 mM to 0.45 mM has been shown to almost entirely turn off PEP consumption [58]. This rapid response enabled accumulation of PEP for the rapid import of glucose when it becomes available again. Using the present framework, we sampled the complex regulatory behaviour using the mechanistic information depicted in Fig. 6B-C. For the regulatory mechanism, however, we also considered the tense form to be capable of performing the reaction, although with a lower activity compared to the relaxed form. Indeed, the model of Smith et al. [57] can be regarded as a special case of our parameterization with *a* ^ref^ = 0. In this case, we are interested in describing the ultrasensitive response of PEPC in the presence/absence of acetyl-CoA under changing FBP concentrations. To this end, experimental data from Xu et al. [58] was used as reference data to build and sample feasible kinetics. The thermodynamic reference state was chosen to ensure initial velocity as done for the mammalian glucokinase. To assess the performance of our framework, we compared our results against an empirical model developed by Lee et al. [59] and calibrated using data collected under similar conditions [55]. The empirical model was adjusted to the same reference state used during sampling to ensure a fair comparison.

**Fig 7:**
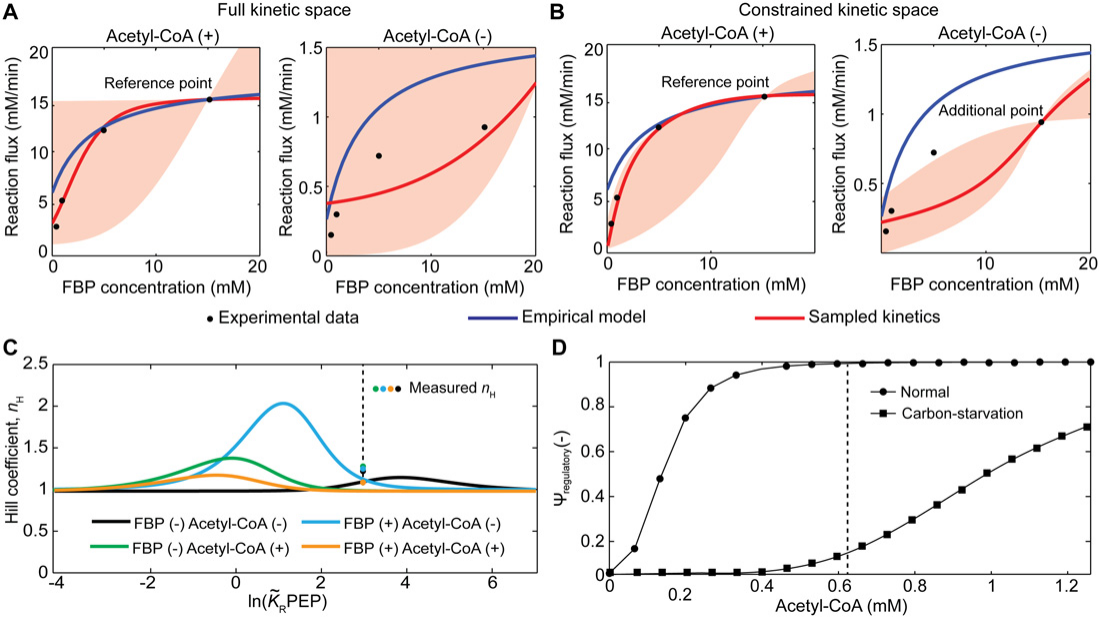
Modelling the regulatory co-activation behaviour of PEPC upon FBP and Acetyl-CoA binding. (A) Comparison of the kinetic behaviour described by the empirical model of Lee et al. [59] (blue line) and the best sampled kinetics using our framework (red line) with and without Acetyl-CoA. The substrates concentrations used in each case were: 2 mM PEP, 10 mM bicarbonate, 15 mM FBP and 0.63 mM acetyl-CoA, which correspond to the physiological concentrations found in *E*. *coli* in glucose-limited cultures [58]. The red shaded area denotes a 99% confidence region for 10^4^ instances sampled. (B) Comparison of the kinetic behaviours including additional information during the sampling. The legends and concentrations used are the same as in (A). (C) Hill coefficient curves for the most accurate PEPC model sampled in the presence and absence of FBP and acetyl-CoA at physiological concentrations. The coloured circles denote experimentally determined Hill coefficients under conditions resembling physiological concentrations of PEP, FBP and acetyl-CoA [55]. (D) Regulatory kinetic behaviour described by the PEPC model with 15 mM of FBP (black circles), *i*.*e*. physiological condition, and 0.45 mM of FBP (black squares), *i*.*e*. carbon-starvation condition, for different acetyl-CoA concentrations. The dashed line represents the physiological concentration of 0.63 mM Acetyl-CoA found in *E*. *coli* under normal physiological conditions.

Our sampling strategy accurately described the kinetic behaviour of the PEPC for different FBP concentrations in the presence of acetyl-CoA at physiological concentrations (Fig. 7A). Moreover, it exhibited a slightly better performance than the model of Lee et al. [59] under the same condition. However, a worse performance of our approach is observed in the absence of acetyl-CoA (Fig. 7A). This was expected as our framework builds kinetic models around one reference state, which in this case was set to 0.63 mM acetyl-CoA and 2 mM PEP. When acetyl-CoA is absent, a larger diversity of plausible kinetics is predicted by our sampling approach, although displaying a more sigmoidal behaviour. In order to improve the fitting to this condition, we can perform a rejection step during the sampling so that every accepted parameter set agrees with the experimental data under this condition. This strategy is typically used in Bayesian Inference by Approximate Bayesian Computation (ABC) methods [60]. In particular, the above method corresponds to the simplest ABC method, the rejection sampler [61]. There are other more efficient samplers implemented within the ABC setting to compute the parameters’ distributions [62,63]; however we opted for the simplest as a-proof-of-principle to demonstrate our strategy. As it can be observed in Fig. 7B, the inclusion of additional experimental data further constraints the plausible kinetic space. Interestingly, even with the addition of extra information during the sampling, the most accurate description for this kinetics displays a sigmoidal behaviour as opposed to the observed hyperbolic kinetics. Indeed, PEPC activation is a fairly complex phenomenon and involves the synergistic interplay of two classes of effectors (type I, *e*.*g*. FBP, and type II, *e*.*g*. acetyl-CoA) [57]. In particular, PEPC activation can be achieved by the sole action of FBP or combined with acetyl-CoA. This behaviour has been previously attributed to play a key role in the rapid adaptation of *E*. *coli* from normal-growing culture conditions to carbon-starvation or acetate switch conditions [58].

To explore this feature more thoroughly, we generated the Hill curves for PEPC upon PEP binding using the most accurate sampled parameter set in the presence/absence of FBP and acetyl-CoA upon binding of PEP (Fig. 7C, see S1 Text for generation of Hill curves). In order to compare our results, we also show experimentally measured Hill coefficients reported by Izui et al. [55] obtained under similar conditions. Firstly, our results are consistent with the theory as they show bell-shaped Hill curves reaching an asymptotic value of unity at either PEP → 0 or PEP → ∞ [64]. Furthermore, they suggest acetyl-CoA is the most powerful activator. As the curves move to the left, the apparent affinity constants increase. The largest displacements are observed in the presence of acetyl-CoA, which supports its role as an invariant activator for the synergistic co-activation of PEPC [57]. Another consequence of the latter is the increase of the enzyme forms in the relaxed state. As the apparent affinity increases, more enzyme forms transition from the tense to the relaxed state. In terms of the prediction of the cooperativity under the different conditions studied, the sampled kinetics is in close agreement with the experimental data. Notably, a theoretical higher cooperativity for PEPC upon PEP binding is observed in the presence of FBP rather than acetyl-CoA (max(*n*_H,Acetyl-CoA(-),FBP(+)_) = 2.05, max(*n*_H,Acetyl-CoA(+)FBP(-)_) = 1.4) (Fig. 7C). These results agree with previous reports indicating low cooperativity for acetyl-CoA alone (1.07< *n*_H_ < 1.2 for 0.02 < acetyl-CoA < 1.0 mM and 0.1 < PEP < 50 mM [55,57]), whereas significant cooperativity for the FBP interaction [53,55,65]. Moreover, our results predict a maximal Hill coefficient of approx. 1.15 for PEPC in the absence of both FBP and acetyl-CoA which is close to the reported value of 1.2 reported by Izui et al. [55].

To further understand the synergistic contributions of the FBP and acetyl-CoA on the PEPC, we explored the impact of acetyl-CoA on the regulatory function (Ψ_regulatory_) using again the most experimentally consistent sampled parameter set. Under carbon-starvation conditions (low FBP) and at acetyl-CoA physiological concentrations, PEPC was only activated in ~10% (Fig. 7D). The presence of both effectors at physiological concentrations as seen in glucose-limited cultures enhanced PEPC activity to almost 99%. Altogether, our results predict a drop of ~90% in the activity of PEPC upon shifting from normal culture conditions to carbon starvation. Experimental values determined for this enzyme simulating these conditions yielded an approx. 94% decrease in its activity [58], close to the one predicted by our model. More importantly, the employed parameterization provides the means to understand how this enzyme is being regulated. As such, this framework is not only capable of sampling and modelling complex kinetic behaviours, but it also provides useful insights into the regulatory mechanisms underpinning those kinetics.

## Discussion

We have presented a general framework for parameterizing and sampling almost any reaction kinetics. Parameterization relies on the integration of the generalized MWC model for modelling the kinetics of oligomeric enzymes with the elementary reaction formalism for deriving the catalytic rates and thermodynamic constraints between rate parameters. As a result, this framework enables exploration of the feasible kinetic space of models following a particular reaction mechanism, displaying a given reference flux under a specific thermodynamic condition. Exploration of the kinetic space in this way provided the necessary tools for evaluating the impact of thermodynamics on enzyme kinetics, as well as the consequences of particular regulatory features on the kinetic behaviour.

Uniform sampling of the kinetic space of reactions enabled inspection of energetic contributions for different reaction molecularities. To that end, a simple relation between conversion terms (catalytic constants) and saturation terms (dissociation constants) was derived. As expected, higher molecularities increase the relative importance of the saturation term on average compared to the conversion term. The latter suggest that higher impacts on the catalysis of multi-substrate enzymes would be expected by modifying its ligand binding and releasing properties rather than the conversion rate of substrates into products. In fact, most common approaches for multi-substrate enzyme engineering involve modifying substrate specificity and selectivity [66], although the success of these strategies will ultimately depend on the particular reaction mechanism and enzyme. Notably, our approach could be employed to sample the rate constants of a particular reaction and determine the flux control coefficient distributions of each step in the mechanism [67]. The latter would provide a broader picture of the pattern regulation, enabling the identification of rate-limiting steps whose modification would most likely improve desired kinetic properties.

This framework was also useful for revealing the impact of the thermodynamic reference state on enzyme kinetics. It has been shown that the reaction elasticities strongly depend on this variable. Our analysis distinguished three elasticity regions as a function of the thermodynamic affinity independent of the bimolecular mechanism analysed: a linear sensitivity region with a steep slope (0> Δ*G*_*r*_>-2 kJ/mol), a transition region (-2> Δ*G*_*r*_>-20 kJ/mol) and a constant sensitivity region (Δ*G*_*r*_<-20 kJ/mol). Notably, substrate and product elasticities reached their highest absolute values close to equilibrium, which agrees with the tendency of substrate elasticities to approach infinity at equilibrium [2]. This framework also enabled analysis far from equilibrium. In all reaction mechanism tested, both substrate and product elasticities reached a plateau, reflecting the saturation state of the enzyme. In terms of the reaction mechanisms, all of them displayed elasticities across the thermodynamic reference states, although a particular behaviour could be distinguished for the random mechanism. The latter suggests that knowledge of the thermodynamic state can be more valuable than exact determination of the reaction mechanism at least for non-random reaction mechanisms.

Exploration of complex kinetic behaviours can also be achieved by employing this framework. To illustrate this, we sampled the kinetic behaviours of the mammalian glucokinase and the phosphoenolpyruvate carboxylase of *E*. *coli*. In the case of the mammalian glucokinase, we were able to model its positive cooperativity for glucose by employing the mechanistic mnemonic model. Random sampling of the kinetic behaviour for this enzyme showed that the experimental Hill coefficient for this enzyme lies surprisingly fairly close to the average Hill coefficient sampled, highlighting the importance of the architecture of its reaction mechanism. Moreover, detailed analysis of the rate constants for the transition from the low- to the high- affinity state confirmed that positive cooperativity for glucose is the result of a slow transition between these two states. In particular, application of our framework to other monomeric proteins exhibiting allosteric behaviours, *e*.*g*. thrombin, myoglobin [68,69], might be a valuable tool to better understand the mechanisms underpinning their kinetics. In the case of PEPC, exploration of the kinetic space provided valuable information related to its regulation. Not only were we able to describe the complex co-activation behaviour of this enzyme by FBP and acetyl-CoA, but also to determine the overall impact of this co-activation on its cooperativity for PEP. Furthermore, our strategy offered insights into the ultrasensitive regulation of PEPC upon binding of FBP and acetyl-CoA. As in the case of mammalian glucokinase, our results emphasises the importance of using mechanistic/phenomenological models for describing and interpreting kinetic behaviours.

We have shown a diverse set of examples illustrating the capabilities of this framework; however our approach holds a vast number of possible further applications. The sampling of enzymatic reactions within metabolic pathways seems particularly promising. The difficulty of parameterizing metabolic pathways is widely recognised with the main difficulty being the large number of parameters and the relative few data available. Rather than grossly simplifying kinetics to enable fitting, this work suggests that it is possible to build feasible, accurate kinetic parameterizations with limited data by integrating phenomenological models with efficient sampling techniques. In particular, we believe that experimentalists could greatly benefit from this framework in those cases where the fitting is difficult or requires large amounts of data. It will be therefore our next step to deploy GRASP as a software package to assist in such complicated tasks.

## Supporting Information

S1 Table Definition of kinetic constants in terms of rate constants for the different mechanism analysed. (DOCX)

S1 Text Mathematical derivations of the formalism, kinetic and thermodynamic expressions. (DOCX)
