# Supplementary material for: A General Framework for Thermodynamically Consistent Parameterization and Efficient Sampling of Enzymatic Reactions
Source: PLoS Comput Biol. 2015 Apr 14;11(4):e1004195. doi: 10.1371/journal.pcbi.1004195 (PMC4397067; doi:10.1371/journal.pcbi.1004195)
Supplement: S1 Table — (DOCX) [file pcbi.1004195.s001.docx]

S1 Table. Definition of kinetic constants in terms of rate constants for the different mechanism analysed

| Kinetic constant | Uni-Uni (*A↔P*)^a^ | Bi-Bi (*A+B↔P+Q*) | Ter-Ter (*A+B+C↔P+Q+R*) |
| --- | --- | --- | --- |
| $\tilde{k}_{\text{cat,+}}$ | $\frac{\tilde{k}_{2}\tilde{k}_{3}}{\tilde{k}_{2}+\tilde{k}_{-2}+\tilde{k}_{3}}$ | $\frac{\tilde{k}_{3}\tilde{k}_{4}\tilde{k}_{5}}{\tilde{k}_{-3}\tilde{k}_{5}+\tilde{k}_{4}\tilde{k}_{5}+\tilde{k}_{3}\tilde{k}_{5}+\tilde{k}_{3}\tilde{k}_{4}}$ | $\frac{\tilde{k}_{4}\tilde{k}_{5}\tilde{k}_{6}\tilde{k}_{7}}{\tilde{k}_{-4}\tilde{k}_{6}\tilde{k}_{7}+\tilde{k}_{5}\tilde{k}_{6}\tilde{k}_{7}+\tilde{k}_{4}\tilde{k}_{6}\tilde{k}_{7}+\tilde{k}_{4}\tilde{k}_{5}\tilde{k}_{7}+\tilde{k}_{4}\tilde{k}_{5}\tilde{k}_{6}}$ |
| $\tilde{k}_{\text{cat,-}}$ | $\frac{\tilde{k}_{-1}\tilde{k}_{-2}}{\tilde{k}_{-1}+\tilde{k}_{2}+\tilde{k}_{-2}}$ | $\frac{\tilde{k}_{-1}\tilde{k}_{-2}\tilde{k}_{-3}}{\tilde{k}_{-2}\tilde{k}_{-3}+\tilde{k}_{-1}\tilde{k}_{-3}+\tilde{k}_{-1}\tilde{k}_{3}+\tilde{k}_{-1}\tilde{k}_{-2}}$ | $\frac{\tilde{k}_{-1}\tilde{k}_{-2}\tilde{k}_{-3}\tilde{k}_{-4}}{\tilde{k}_{-2}\tilde{k}_{-3}\tilde{k}_{-4}+\tilde{k}_{-1}\tilde{k}_{-3}\tilde{k}_{-4}+\tilde{k}_{-1}\tilde{k}_{-2}\tilde{k}_{-4}+\tilde{k}_{-1}\tilde{k}_{-2}\tilde{k}_{-3}+\tilde{k}_{-1}\tilde{k}_{-2}\tilde{k}_{4}}$ |
| $\tilde{K}_{\text{(A)}}$ | $\frac{\tilde{k}_{-1}\tilde{k}_{-2}+\tilde{k}_{-1}\tilde{k}_{3}+\tilde{k}_{2}\tilde{k}_{3}}{\tilde{k}_{1}\left(\tilde{k}_{2}+\tilde{k}_{-2}+\tilde{k}_{3}\right)}$ | $\frac{\tilde{k}_{-1}}{\tilde{k}_{1}}$ | $\frac{\tilde{k}_{-1}}{\tilde{k}_{1}}$ |
| $\tilde{K}_{\text{(P)}}$ | $\frac{\tilde{k}_{-1}\tilde{k}_{-2}+\tilde{k}_{-1}\tilde{k}_{3}+\tilde{k}_{2}\tilde{k}_{3}}{\tilde{k}_{-3}\left(\tilde{k}_{-1}+\tilde{k}_{2}+\tilde{k}_{-2}\right)}$ | $\frac{\tilde{k}_{-1}\left(\tilde{k}_{-2}\tilde{k}_{-3}+\tilde{k}_{-2}\tilde{k}_{4}+\tilde{k}_{3}\tilde{k}_{4}\right)}{\tilde{k}_{-4}\left(\tilde{k}_{-2}\tilde{k}_{-3}+\tilde{k}_{-1}\tilde{k}_{-3}+\tilde{k}_{-1}\tilde{k}_{3}+\tilde{k}_{-1}\tilde{k}_{-2}\right)}$ | $\frac{\tilde{k}_{-1}\tilde{k}_{-2}\left(\tilde{k}_{-3}\tilde{k}_{-4}+\tilde{k}_{-3}\tilde{k}_{5}+\tilde{k}_{4}\tilde{k}_{5}\right)}{\tilde{k}_{-5}\left(\tilde{k}_{-2}\tilde{k}_{-3}\tilde{k}_{-4}+\tilde{k}_{-1}\tilde{k}_{-3}\tilde{k}_{-4}+\tilde{k}_{-1}\tilde{k}_{-2}\tilde{k}_{-4}+\tilde{k}_{-1}\tilde{k}_{-2}\tilde{k}_{-3}+\tilde{k}_{-1}\tilde{k}_{-2}\tilde{k}_{4}\right)}$ |
| $\tilde{K}_{\text{(B)}}$ |  | $\frac{\tilde{k}_{5}\left(\tilde{k}_{-2}\tilde{k}_{-3}+\tilde{k}_{-2}\tilde{k}_{4}+\tilde{k}_{3}\tilde{k}_{4}\right)}{\tilde{k}_{2}\left(\tilde{k}_{-3}\tilde{k}_{5}+\tilde{k}_{4}\tilde{k}_{5}+\tilde{k}_{3}\tilde{k}_{5}+\tilde{k}_{3}\tilde{k}_{4}\right)}$ | $\frac{\tilde{k}_{-2}}{\tilde{k}_{2}}$ |
| $\tilde{K}_{\text{(Q)}}$ |  | $\frac{\tilde{k}_{5}}{\tilde{k}_{-5}}$ | $\frac{\tilde{k}_{6}}{\tilde{k}_{-6}}$ |
| $\tilde{K}_{\text{(C)}}$ |  |  | $\frac{\tilde{k}_{6}\tilde{k}_{7}\left(\tilde{k}_{-3}\tilde{k}_{-4}+\tilde{k}_{-3}\tilde{k}_{5}+\tilde{k}_{4}\tilde{k}_{5}\right)}{\tilde{k}_{3}\left(\tilde{k}_{-4}\tilde{k}_{6}\tilde{k}_{7}+\tilde{k}_{5}\tilde{k}_{6}\tilde{k}_{7}+\tilde{k}_{4}\tilde{k}_{6}\tilde{k}_{7}+\tilde{k}_{4}\tilde{k}_{5}\tilde{k}_{7}+\tilde{k}_{4}\tilde{k}_{5}\tilde{k}_{6}\right)}$ |
| $\tilde{K}_{\text{(R)}}$ |  |  | $\frac{\tilde{k}_{7}}{\tilde{k}_{-7}}$ |

^a^ Expressions inside the parenthesis represent the overall reaction for the mechanism analysed.
