# Supplementary material for: A General Framework for Thermodynamically Consistent Parameterization and Efficient Sampling of Enzymatic Reactions
Source: PLoS Comput Biol. 2015 Apr 14;11(4):e1004195. doi: 10.1371/journal.pcbi.1004195 (PMC4397067; doi:10.1371/journal.pcbi.1004195)
Supplement: S1 Text — (DOCX) [file pcbi.1004195.s002.docx]

**S1 Text. Mathematical derivations of the formalism, kinetic and thermodynamic expressions**

General framework for thermodynamically consistent parameterization and efficient sampling of enzymatic reactions

Pedro Saa, Lars K. Nielsen

**Table of Contents**

[1. Monod-Wyman-Changeux allosteric model 2](#_Toc408396151)

[2. Generalization of the principle of microscopic reversibility under non-equilibrium condition 4](#_Toc408396152)

[3. Derivation of the general expression for determination of rate constants 7](#_Toc408396153)

[4. Modified Haldane relationships under non-equilibrium steady-state condition 11](#_Toc408396154)

[5. Analysis of reaction elasticities near and far from equilibrium 13](#_Toc408396155)

[5.1. Preliminary definitions 13](#_Toc408396156)

[5.2. Near equilibrium elasticity analysis 14](#_Toc408396157)

[5.3. Far from equilibrium elasticity analysis 14](#_Toc408396158)

[6. Calculation of Hill curves 16](#_Toc408396159)

[References 17](#_Toc408396160)

1. Monod-Wyman-Changeux allosteric model

The MWC model is used for modeling complex reactions catalysed by allosteric enzymes. Primarily, the MWC framework rests in the following assumptions [[1](#_ENREF_1)]:

- Allosteric proteins are oligomers containing identical subunits and all these allosteric subunits occupy equivalent positions, axial and rotational symmetry.
- There is only one stereospecific ligand binding site on each allosteric subunit or *protomer.*
- The conformation of each of these protomers is constrained by its association with the other protomers.
- At least two states are reversibly accessible to the whole system and dependent on inter-subunit bonds (concerted model).
- Transition from one to the other state alters the conformation and the affinity of the protomers’ binding sites for the corresponding ligand.
- When the enzyme goes from one state to another state, its molecular symmetry is conserved.

Assuming that the two conformational states of the enzyme are in equilibrium, application of the above statements leads to the derivation of *two independent functions* describing the behaviour of an oligomeric enzyme with *n* protomers, namely: (1) the fraction of protein in the R state $\left(\bar{R}\right)$, and (2) the fraction of sites actually bound by the ligand $\left(\bar{Y}\right)$.

$\bar{R}=\frac{R_{0}+R_{1}+...+R_{n}}{(R_{0}+R_{1}+...+R_{n})+(T_{0}+T_{1}+...+T_{n})}=\frac{(1+\alpha)^{n}}{L(1+c\alpha)^{n}+(1+\alpha)^{n}}$ (S1)

$\bar{Y}=\frac{\left(R_{1}+2R_{2}+...+nR_{n}\right)+\left(T_{1}+2T_{2}+...+nT_{n}\right)}{n\left[(R_{0}+R_{1}+...+R_{n})+(T_{0}+T_{1}+...+T_{n})\right]}=\frac{Lc\alpha(1+c\alpha)^{n-1}+\alpha(1+\alpha)^{n-1}}{L(1+c\alpha)^{n}+(1+\alpha)^{n}}$ (S2)

In Eqs. S1-2, *L* denotes the equilibrium constant (also denoted the allosteric constant) for the $R_{0}\leftrightarrow T_{0}$ transition, $\alpha$ represents the ligand saturation calculated as the ratio of the ligand concentration and the dissociation constant for the R state ($K_{\text{R}}$) and *c* denotes the ratio of the dissociation constants for the relaxed and tense states $\left(c=K_{\text{R}}/K_{\text{T}}\right)$. The MCW model states that cooperativity (homotropic effects) results out of the equilibrium shift from the T to the R state upon ligand binding, which ultimately depends on the values of *L* and *c*. On the other hand, allosteric interactions (heterotrophic effects) can be accounted for by noting that allosteric effectors displace the spontaneous equilibrium between the R and T states of the enzyme, *i.e.* they modulate the allosteric constant. Although there are exceptions for many of the aforementioned assumptions, *e.g.* many receptors have non-identical subunits [[2](#_ENREF_2),[3](#_ENREF_3)], monomers can have primary and secondary binding sites [[4](#_ENREF_4)], among others, Eqs. S1-2 provide the basis for modeling most cooperative and allosteric behaviours (excluding negative cooperativity) [[1](#_ENREF_1)].

1. Generalization of the principle of microscopic reversibility under non-equilibrium condition

We shall show that formulation of all fundamental cycles leads to the general expression shown in Eq. 10. As an example, let us consider the following branched mechanism,


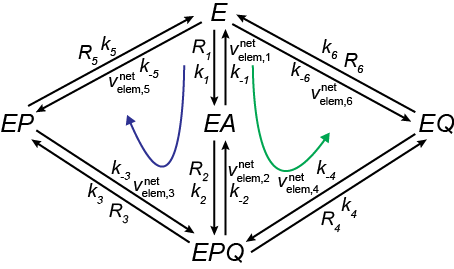


**Fig. S1 Illustration of a random Uni-Bi random reaction mechanism.** In each step of the mechanism, the respective reversibility, elementary net flux and forward and reverse constants are shown. There are 2 feasible cycles converting *A* ↔ *P* + *Q*, namely: *E → EA → EPQ→ EP → E* (blue cycle) and *E → EA → EPQ → EQ → E* (green cycle)

This mechanism has two cycles able of carrying out the overall reaction *A↔P+Q*. At equilibrium, the ratio of the product of forward rate constants to the product of the reverse rate constants yields the equilibrium constant of the reaction. In this case, the blue and green cycles must obey this relation as they catalyse the same reaction. By employing this condition, we are implicitly imposing the principle of microscopic reversibility for this reaction.

$K_{\text{eq}}=\mathop{\frac{k_{1}k_{2}k_{3}k_{5}}{k_{-1}k_{-2}k_{-3}k_{-5}}}\limits_{\text{blue cycle}} =\mathop{\frac{k_{1}k_{2}k_{4}k_{6}}{k_{-1}k_{-2}k_{-4}k_{-6}}}\limits_{\text{green cycle}}$ (S3)

In the following, we will work with the blue cycle as the demonstration is analogous for the green cycle. By using the scaling procedure described in Eq. 6 for each rate constant, *i.e.* $k_{i}=\tilde{k}_{i}/\left(x_{i}\cdot e_{\text{total}}\right)$, the previous equation takes the following form,

$K_{\text{eq}}=\frac{\tilde{k}_{1}\tilde{k}_{2}\tilde{k}_{3}\tilde{k}_{5}}{\tilde{k}_{-1}\tilde{k}_{-2}\tilde{k}_{-3}\tilde{k}_{-5}}\cdot\frac{P^{\mathrm{ref}}Q^{\mathrm{ref}}}{A^{\mathrm{ref}}}$ (S4)

where *P*^ref^*Q*^ref^/*A*^ref^ represents the ratio of products to substrates (mass-action ratio) at the reference steady-state. By applying logarithm to this equation and employing the definition of the Gibbs free energy difference of reaction, Eq. S4 can be expressed as follows,

$\begin{matrix} \ln\left(K_{\text{eq}}\right)=\ln\left(\frac{\tilde{k}_{1}\tilde{k}_{2}\tilde{k}_{3}\tilde{k}_{5}}{\tilde{k}_{-1}\tilde{k}_{-2}\tilde{k}_{-3}\tilde{k}_{-5}}\right)+\ln\left(\frac{P^{\mathrm{ref}}Q^{\mathrm{ref}}}{A^{\mathrm{ref}}}\right) \\ -\ln\left(K_{\text{eq}}\right)+\ln\left(\frac{P^{\mathrm{ref}}Q^{\mathrm{ref}}}{A^{\mathrm{ref}}}\right)=\ln\left(\frac{\tilde{k}_{1}\tilde{k}_{2}\tilde{k}_{3}\tilde{k}_{5}}{\tilde{k}_{-1}\tilde{k}_{-2}\tilde{k}_{-3}\tilde{k}_{-5}}\right) \\ \frac{\Delta G_{r}^{o}}{RT}+\ln\left(\frac{P^{\mathrm{ref}}Q^{\mathrm{ref}}}{A^{\mathrm{ref}}}\right)=\ln\left(\frac{\tilde{k}_{1}\tilde{k}_{2}\tilde{k}_{3}\tilde{k}_{5}}{\tilde{k}_{-1}\tilde{k}_{-2}\tilde{k}_{-3}\tilde{k}_{-5}}\right) \\ \frac{\Delta G_{r}}{RT}=\ln\left(\frac{\tilde{k}_{1}\tilde{k}_{2}\tilde{k}_{3}\tilde{k}_{5}}{\tilde{k}_{-1}\tilde{k}_{-2}\tilde{k}_{-3}\tilde{k}_{-5}}\right) \end{matrix}$ (S5)

On the other hand, each elementary flux can be written in terms of the respective reversibility and scaled rate constant using Eq. 9,

$\begin{matrix} ln\left(R_{i}\right)=sgn\left(v^{\text{ref}}\right)\cdot\text{ln}\left(\frac{v_{\text{elem,}i}^{\text{reverse}}}{v_{\text{elem,}i}^{\text{forward}}}\right) \\ ln\left(R_{i}\right)=sgn\left(v^{\text{ref}}\right)\cdot\text{ln}\left(\frac{\tilde{k}_{-i}\cdot\tilde{x}_{\text{elem,}i}^{\text{reverse}}\cdot\tilde{e}_{\text{elem,}i}^{\text{reverse}}}{\tilde{k}_{i}\cdot\tilde{x}_{\text{elem,}i}^{\text{forward}}\cdot\tilde{e}_{\text{elem,}i}^{\text{forward}}}\right) \end{matrix}$ (S6)

Taking the sum for all the reversibilities contained in the blue cycle we obtain,

$\begin{matrix} ln\left(R_{i}\right)=sgn\left(v^{\text{ref}}\right)\cdot\text{ln}\left(\frac{v_{\text{elem,}i}^{\text{reverse}}}{v_{\text{elem,}i}^{\text{forward}}}\right) \\ \sum_{i=\left\{1,2,3,5\right\}} ln\left(R_{i}\right)=sgn\left(v^{\text{ref}}\right)\cdot\left(\begin{matrix} \sum_{i=\left\{1,2,3,5\right\}} \text{ln}\left(\frac{\tilde{k}_{-i}}{\tilde{k}_{i}}\right)+\sum_{i=\left\{1,2,3,5\right\}} \text{ln}\left(\tilde{x}_{\text{elem,}i}^{\text{reverse}}\right) \\ -\sum_{i=\left\{1,2,3,5\right\}} \text{ln}\left(\tilde{x}_{\text{elem,}i}^{\text{forward}}\right)+\sum_{i=\left\{1,2,3,5\right\}} \text{ln}\left(\tilde{e}_{\text{elem,}i}^{\text{reverse}}\right)-\sum_{i=\left\{1,2,3,5\right\}} \text{ln}\left(\tilde{e}_{\text{elem,}i}^{\text{forward}}\right) \end{matrix}\right) \end{matrix}$ (S7)

Notably, the sum of the logarithms of the scaled metabolite and enzyme intermediates yields the same result in both the reverse and forward directions, as we are traversing the same cycle. As such, we are left with the following simple expression,

$\sum_{i=\left\{1,2,3,5\right\}} ln\left(R_{i}\right)=sgn\left(v^{\text{ref}}\right)\cdot\text{ln}\left(\frac{\tilde{k}_{1}\tilde{k}_{2}\tilde{k}_{3}\tilde{k}_{5}}{\tilde{k}_{-1}\tilde{k}_{-2}\tilde{k}_{-3}\tilde{k}_{-5}}\right)$ (S8)

Substituting the expression in the right-hand side of Eq. S8 with the expression previously derived in Eq. S5 yields,

$\sum_{i=\left\{1,2,3,5\right\}} ln\left(R_{i}\right)=sgn\left(v^{\text{ref}}\right)\cdot\frac{\Delta G_{r}}{RT}$ (S9)

Similarly, it can be derived the analogous expression for the green cycle,

$\sum_{i=\left\{1,2,4,6\right\}} ln\left(R_{i}\right)=sgn\left(v^{\text{ref}}\right)\cdot\frac{\Delta G_{r}}{RT}$ (S10)

Altogether, Eqs. S9 and S10 can be cast in the general form described by Eq. 10 as follows,

$\mathop{\sum\ln(R_{i})}\limits_{i\in\text{fundamental cycle}} =\mathrm{sgn}\left(v^{\text{ref}}\right)\cdot\frac{\Delta G_{r}}{RT}$ (S11)

The previous demonstration can be generalized to any other mechanism provided that it initially starts by finding all the fundamental cycles in the mechanism. It has been demonstrated that formulation of all fundamental cycles ensures obeying the principle of microscopic reversibility. For proofs and details of the latter, we direct the reader to Colquhuon et al. [[5](#_ENREF_5)].

1. Derivation of the general expression for determination of rate constants

As previously mentioned, calculation of the rate constants can be achieved using the general Eq. 17. In this section, we will now show how to derive this expression using the same example from the previous section. In this mechanism, there are 12 elementary mass balances considering both the forward and reverse directions. The complete set of mass balances for the forward fluxes is given by Eqs. S12, whereas for the reverse fluxes Eqs. S13 describe the respective balances,

$\begin{matrix} v_{\text{elem,1}}^{\text{ref}}=\tilde{k}_{1}\cdot\tilde{e}^{\text{ref}} \\ v_{\text{elem,2}}^{\text{ref}}=\tilde{k}_{2}\cdot\tilde{e}a^{\text{ref}}\text{ } \\ v_{\text{elem,3}}^{\text{ref}}=\tilde{k}_{3}\cdot\tilde{e}pq^{\text{ref}} \\ v_{\text{elem,4}}^{\text{ref}}=\tilde{k}_{4}\cdot\tilde{e}pq^{\text{ref}} \\ v_{\text{elem,5}}^{\text{ref}}=\tilde{k}_{5}\cdot\tilde{e}p^{\text{ref}} \\ v_{\text{elem,6}}^{\text{ref}}=\tilde{k}_{6}\cdot\tilde{e}q^{\text{ref}} \end{matrix}$ (S12)

$\begin{matrix} v_{\text{elem,-1}}^{\text{ref}}=\tilde{k}_{-1}\cdot\tilde{e}a^{\text{ref}} \\ v_{\text{elem,-2}}^{\text{ref}}=\tilde{k}_{-2}\cdot\tilde{e}pq^{\text{ref}} \\ v_{\text{elem,-3}}^{\text{ref}}=\tilde{k}_{-3}\cdot\tilde{e}p^{\text{ref}} \\ v_{\text{elem,-4}}^{\text{ref}}=\tilde{k}_{-4}\cdot\tilde{e}q^{\text{ref}} \\ v_{\text{elem,-5}}^{\text{ref}}=\tilde{k}_{-5}\cdot\tilde{e}^{\text{ref}} \\ v_{\text{elem,-6}}^{\text{ref}}=\tilde{k}_{-6}\cdot\tilde{e}^{\text{ref}} \end{matrix}$ (S13)

It can be seen that both balances sets can be cast in matrix form by carefully considering the enzyme intermediates involved in each elementary mass balance. We can rewrite these two sets of equations in the following generic format as shown in Eq. 7,

$\begin{matrix} v_{\text{elem,+}}^{\text{ref}}=P_{+}\text{(}\tilde{e}^{\text{ref}}\text{)}\cdot\tilde{k}_{+} \\ v_{\text{elem,}-}^{\text{ref}}=P_{-}\text{(}\tilde{e}^{\text{ref}}\text{)}\cdot\tilde{k}_{-} \end{matrix}$ (S14)

where the subscripts + and – denote the balances for the forward and reverse elementary fluxes, respectively. In both cases, the ***P*** matrix denotes a diagonal matrix containing the appropriate scaled enzyme intermediate abundance for each mass balance. This matrix only depends on the particular reaction mechanism. Following this notation, the elements in Eqs. S14 can be defined as follows,

$\begin{matrix} P_{+}=\left[\begin{matrix} \tilde{e}^{\text{ref}} & 0 & 0 & 0 & 0 & 0 \\ 0 & \tilde{e}a^{\text{ref}} & 0 & 0 & 0 & 0 \\ 0 & 0 & \tilde{e}pq^{\text{ref}} & 0 & 0 & 0 \\ 0 & 0 & 0 & \tilde{e}pq^{\text{ref}} & 0 & 0 \\ 0 & 0 & 0 & 0 & \tilde{e}p^{\text{ref}} & 0 \\ 0 & 0 & 0 & 0 & 0 & \tilde{e}q^{\text{ref}} \end{matrix}\right]\text{ }P_{-}=\left[\begin{matrix} \tilde{e}a^{\text{ref}} & 0 & 0 & 0 & 0 & 0 \\ 0 & \tilde{e}pq^{\text{ref}} & 0 & 0 & 0 & 0 \\ 0 & 0 & \tilde{e}p^{\text{ref}} & 0 & 0 & 0 \\ 0 & 0 & 0 & \tilde{e}q^{\text{ref}} & 0 & 0 \\ 0 & 0 & 0 & 0 & \tilde{e}^{\text{ref}} & 0 \\ 0 & 0 & 0 & 0 & 0 & \tilde{e}^{\text{ref}} \end{matrix}\right]\text{ } \\ \tilde{k}_{+}=\left[\begin{matrix} \tilde{k}_{1} \\ \tilde{k}_{2} \\ \tilde{k}_{3} \\ \tilde{k}_{4} \\ \tilde{k}_{5} \\ \tilde{k}_{6} \end{matrix}\right] \tilde{k}_{-}=\left[\begin{matrix} \tilde{k}_{-1} \\ \tilde{k}_{-2} \\ \tilde{k}_{-3} \\ \tilde{k}_{-4} \\ \tilde{k}_{-5} \\ \tilde{k}_{-6} \end{matrix}\right]\text{ }v_{\text{elem,+}}^{\text{ref}}=\left[\begin{matrix} v_{\text{elem},1}^{\text{ref}} \\ v_{\text{elem},2}^{\text{ref}} \\ v_{\text{elem},3}^{\text{ref}} \\ v_{\text{elem},4}^{\text{ref}} \\ v_{\text{elem},5}^{\text{ref}} \\ v_{\text{elem},6}^{\text{ref}} \end{matrix}\right]\text{ }v_{\text{elem,}-}^{\text{ref}}=\left[\begin{matrix} v_{\text{elem},-1}^{\text{ref}} \\ v_{\text{elem},-2}^{\text{ref}} \\ v_{\text{elem},-3}^{\text{ref}} \\ v_{\text{elem},-4}^{\text{ref}} \\ v_{\text{elem},-5}^{\text{ref}} \\ v_{\text{elem},-6}^{\text{ref}} \end{matrix}\right] \end{matrix}$(S15)

In order to find the rate constants, we still need to formulate additional relations that enable solving each elementary flux. These relations can be derived from the reversibilities and knowledge of the reaction flux [[6](#_ENREF_6)]. For each linear step in the mechanism, the difference of the forward and reverse flux has to yield the reference reaction flux (*v*^ref^), whereas at the branching node *EPQ* (Figure 1S) the sum of the differences of the forward and reverse fluxes for each branch has to be equal to the reference flux. At this point, it is convenient to define the pattern stoichiometry (***S***_pattern_) in terms of the difference between the elementary reaction fluxes $\left(v_{\text{elem,}i}^{\text{net}}\right)$ as this facilitates calculations. At steady-state, the elementary net fluxes must satisfy the condition $S_{\text{pattern}}\cdot v_{\text{elem}}^{\text{net}}=0$, where each element is defined as follows,

$S_{\text{pattern}}=\left[\begin{matrix} -1 & 0 & 0 & 0 & 1 & 1 \\ 1 & -1 & 0 & 0 & 0 & 0 \\ 0 & 1 & -1 & -1 & 0 & 0 \\ 0 & 0 & 1 & 0 & -1 & 0 \\ 0 & 0 & 0 & 1 & 0 & -1 \end{matrix}\right]\text{ }v_{\text{elem}}^{\text{net}}=\left[\begin{matrix} v_{\text{elem,1}}^{\text{net}} \\ v_{\text{elem,2}}^{\text{net}} \\ v_{\text{elem,3}}^{\text{net}} \\ v_{\text{elem,4}}^{\text{net}} \\ v_{\text{elem,5}}^{\text{net}} \\ v_{\text{elem,6}}^{\text{net}} \end{matrix}\right]$ (S16)

This stoichiometric matrix is by definition singular as it defines the mass balances within a cycle. Thus, there is always a non-trivial solution satisfying the steady-state condition. An efficient way of solving this condition is expressing the solution space as a linear combination of the null basis ***N***_pattern_. We can next uniformly sample the weights (***w***) that yield a non-negative solution with maximum elementary net flux *v*^ref^. As such, we can find the branching structure of the pattern as a function of the vector ***w*** subject to the previous constraints,

$r_{\text{elem}}(w)=N_{\text{pattern}}\cdot w$ (S17)

where each component is defined as,

$N_{\text{pattern}}=\left[\begin{matrix} 1 & 1 \\ 1 & 1 \\ 1 & 1 \\ 0 & 1 \\ 1 & 0 \\ 0 & 1 \end{matrix}\right]\text{ , }w=\left[\begin{matrix} w_{1} \\ w_{2} \end{matrix}\right]$ (S18)

Notably, the weights *w*_1_ and *w*_2_ represent how much flux is being diverted to each of the mechanisms’ branches (Figure 1S). Once we know how much flux is being carried by each elementary step, we can calculate the forward and reverse fluxes using the reversibilities definition (Eq. 9). The ratio of the forward and reverse elementary fluxes is related to the reversibilities as follows,

$\begin{matrix} \Gamma_{i}^{\text{forward}}=\frac{v_{\text{elem},i}^{\text{ref}}}{v_{\text{elem},i}^{\text{net}}}=\frac{v_{\text{elem},i}^{\text{ref}}}{v_{\text{elem},i}^{\text{ref}}-v_{\text{elem},-i}^{\text{ref}}}=\frac{1}{1-v_{\text{elem},-i}^{\text{ref}}/v_{\text{elem},i}^{\text{ref}}}=\frac{1}{1-R_{i}^{sgn\left(v^{\text{ref}}\right)}} \\ \Gamma_{i}^{\text{reverse}}=\frac{v_{\text{elem},-i}^{\text{ref}}}{v_{\text{elem},i}^{\text{net}}}=\frac{v_{\text{elem},-i}^{\text{ref}}}{v_{\text{elem},i}^{\text{ref}}-v_{\text{elem},-i}^{\text{ref}}}=\frac{v_{\text{elem},-i}^{\text{ref}}/v_{\text{elem},i}^{\text{ref}}}{1-v_{\text{elem},-i}^{\text{ref}}/v_{\text{elem},i}^{\text{ref}}}=\frac{R_{i}^{sgn\left(v^{\text{ref}}\right)}}{1-R_{i}^{sgn\left(v^{\text{ref}}\right)}} \end{matrix}$ (S19)

Thus, each elementary flux (forward and reverse) can be computed as the product of respective Γ*_i_* element times the elementary net flux given by the branching vector ***r***_elem_(***w***).

$\begin{matrix} v_{\text{elem,+}}^{\text{ref}}=\Gamma_{+}(R)\cdot r_{\text{elem}}(w) \\ v_{\text{elem,}-}^{\text{ref}}=\Gamma_{-}(R)\cdot r_{\text{elem}}(w) \end{matrix}$ (S20)

Where the **Γ**_+_ and **Γ**- represent diagonal matrices with the respective Γ*_i_* elements. Finally, the forward and reverse rate constant vectors are found by combining Eqs. S14 with S20,

$\begin{matrix} \text{Forward rate constants} \\ P_{+}\text{(}\tilde{e}^{\text{ref}}\text{)}\cdot k_{+}^{\text{ref}}=\Gamma_{+}(R)\cdot r_{\text{elem}}(w) \\ k_{+}^{\text{ref}}=P_{+}^{-1}\text{(}\tilde{e}^{\text{ref}}\text{)}\cdot\Gamma_{+}(R)\cdot r_{\text{elem}}(w) \\ \text{Reverse rate constants} \\ P_{-}\text{(}\tilde{e}^{\text{ref}}\text{)}\cdot k_{-}^{\text{ref}}=\Gamma_{-}(R)\cdot r_{\text{elem}}(w) \\ k_{-}^{\text{ref}}=P_{-}^{-1}\text{(}\tilde{e}^{\text{ref}}\text{)}\cdot\Gamma_{-}(R)\cdot r_{\text{elem}}(w) \end{matrix}$ (S21)

Equations S21 enable calculation of the rate constants for any reaction mechanism as a function of the normalized enzyme intermediate abundances, reversibilities and branching vector. As such, this parameterization is consistent with the reaction thermodynamics, pattern stoichiometry and reaction flux at a reference point.

1. Modified Haldane relationships under non-equilibrium steady-state condition

Without any loss generality it can be shown that within a fundamental cycle, the logarithm of the ratio of the product of the scaled reverse to forward rates is equal to the thermodynamic affinity of the reaction (Eq. S8-9).

$\frac{\Delta G_{r}}{RT}=\mathop{\ln\left(\prod\frac{\tilde{k}_{i,\text{reverse}}}{\tilde{k}_{i,\text{forward}}}\right)}\limits_{i\in\text{fundamental cycle}}$ (S22)

This relation can be always formulated by imposing the principle of microscopic reversibility and employing the definitions of the scaled rate constants. Equation S21 can be further transformed into a more familiar form.

$\exp\left(\frac{\Delta G_{r}}{RT}\right)=\mathop{\prod\frac{\tilde{k}_{i,\text{reverse}}}{\tilde{k}_{i,\text{forward}}}}\limits_{i\in\text{fundamental cycle}}$ (S23)

Remarkably, the right-hand side of Eq. S23 maintains a particular resemblance to the fundamental relation between the ratio of the forward and reverse constants and the equilibrium constant of the reaction, *i.e.* $K_{\text{eq}}=\mathop{\prod\frac{k_{i,\text{forward}}}{k_{i,\mathrm{reverse}}}}\limits_{i\in\text{fundamental cycle}}$. The main differences between these two expressions are that Eq. S23 uses scaled rate constant and that the ratio of constants is inverted. Nevertheless, we can still exploit this similarity and derive Haldane-like relationships under non-equilibrium steady-state conditions. It is always possible to derive the following Haldane relationship for a particular reaction mechanism [[7](#_ENREF_7)],

$K_{\text{eq}}=\frac{k_{\text{cat,+}}}{k_{\text{cat,-}}}\cdot\prod_{i}^{s} \prod_{j}^{p} \frac{K_{j}}{K_{i}}$ (S24)

Where *K_i_* and *K_j_* represent substrate and product dissociation constants, *k*_cat,+_ and *k*_cat,-_are the catalytic constants for the forward and reverse reactions, respectively, and *s* and *p* denote the substrates and products of the reaction. In a similar form, we can derive an analogous expression for Eq. S23 in terms of the scaled constants.

$\exp\left(\frac{\Delta G_{r}}{RT}\right)=\frac{\tilde{k}_{\text{cat,-}}}{\tilde{k}_{\text{cat,+}}}\cdot\prod_{i}^{s} \prod_{j}^{p} \frac{\tilde{K}_{i}}{\tilde{K}_{j}}$ (S25)

By applying logarithm to the previous expression and regrouping terms, we can derive Eq. S25 under non-equilibrium steady-state,

$\frac{\Delta G_{r}}{RT}=\ln\left(\frac{\tilde{k}_{\text{cat,-}}}{\tilde{k}_{\text{cat,+}}}\cdot\prod_{i}^{s} \prod_{j}^{p} \frac{\tilde{K}_{i}}{\tilde{K}_{j}}\right)$ (S26)

$\frac{\Delta G_{r}}{RT}=\mathop{\ln\left(\frac{\tilde{k}_{\text{cat,-}}}{\tilde{k}_{\text{cat,+}}}\right)}\limits_{\text{catalysis}} +\mathop{\sum_{i}^{s} \sum_{j}^{p} \ln\left(\frac{\tilde{K}_{i}}{\tilde{K}_{j}}\right)}\limits_{\text{binding}}$ (S27)

Equation S27 enables individual analysis of the energetic contributions of the scaled catalytic (turnover) and dissociation (binding) constants under different thermodynamic reference conditions and for different reaction mechanisms.

1. Analysis of reaction elasticities near and far from equilibrium
   1. Preliminary definitions

In the following section we will examine the asymptotic behaviour of reaction elasticities near and far from equilibrium. As an illustrative example, we analyse a Uni-Uni reaction described with generic reversible Michaelis-Menten kinetics representing the conversion of a substrate *A* into a product *P*. A similar analysis can be performed on the elasticity behaviour of multi-substrate kinetics; however the derivation is cumbersome and more difficult to interpret.

Let us consider the following rate law describing the above mentioned reaction,

$v=\frac{V_{\max,+}}{K_{A}}\cdot\frac{A-P/K_{\text{eq}}}{1+A/K_{A}+P/K_{P}}$ (S28)

where *V*_max,+_ represents the maximum forward reaction rate, *K_A_* and *K_P_* denote the dissociation constants for the substrate *A* and product *P*, respectively, and *K*_eq_ is the equilibrium constant for the reaction. The substrate and product elasticities for this rate law are given by [[8](#_ENREF_8)],

$\begin{matrix} \varepsilon_{S}^{v}=\frac{S}{v}\frac{\partial v}{\partial S}=\frac{1}{1-\Lambda/K_{\text{eq}}}-\frac{A/K_{A}}{1+A/K_{A}+P/K_{P}} \\ \varepsilon_{P}^{v}=\frac{P}{v}\frac{\partial v}{\partial P}=-\frac{\Lambda/K_{\text{eq}}}{1-\Lambda/K_{\text{eq}}}-\frac{P/K_{P}}{1+A/K_{A}+P/K_{P}} \end{matrix}$ (S29)

where Λ = *P*/*S* represents the mass action ratio. This quantity is related to the Gibbs free energy difference of reaction and *K*_eq_ as follows,

$\begin{matrix} \Delta G_{r}^{'}=\Delta G_{r}^{\circ}+RT\ln\left(P/S\right)\text{ , at equilibrium }\Delta G_{r}^{\circ}=-RT\ln\left(K_{\mathrm{eq}}\right) \\ \Delta G_{r}^{'}=-RT\ln\left(K_{\mathrm{eq}}\right)+RT\ln\left(\Lambda\right) \\ \Delta G_{r}^{'}=RT\ln\left(\Lambda/K_{\mathrm{eq}}\right) \\ \exp\left(\frac{\Delta G_{r}^{'}}{RT}\right)=\frac{\Lambda}{K_{\mathrm{eq}}}=\frac{P}{S\cdot K_{\mathrm{eq}}} \end{matrix}$ (S30)

Equation (S30) relates the thermodynamic driving force of the reaction with the concentration of reactants, thereby establishing a direct link between thermodynamics and kinetics. As it will be seen below, this expression will be of great help to determine the elasticity behaviour near and far from equilibrium.

- 1. Near equilibrium elasticity analysis

We are interested in determining the behaviour of the reaction elasticities when $\Delta G_{r}^{'}\to0$. Let us take the limit of the substrate elasticity close to equilibrium.

$\lim_{\Delta G_{r}^{'}\to0} \varepsilon_{S}^{v}=\lim_{\Delta G_{r}^{'}\to0} \frac{1}{1-\Lambda/K_{\text{eq}}}-\frac{A/K_{A}}{1+A/K_{A}+P/K_{P}}$ (S31)

We can recast Eq. S31 in a more convenient form using Eq. S30 as follows,

$\lim_{\Delta G_{r}^{'}\to0} \varepsilon_{S}^{v}=\lim_{\Delta G_{r}^{'}\to0} \frac{1}{1-\exp\left(\Delta G_{r}^{'}/RT\right)}-\frac{A/K_{A}}{1+A/K_{A}+\exp\left(\Delta G_{r}^{'}/RT\right)\cdot K_{\text{eq}}K_{A}/K_{P}}$ (S32)

When $\Delta G_{r}^{'}\to0$ the denominator of the first term of Eq. S32 approaches zero, thus the limit is undefined approaching +∞. Similarly, the product elasticity approaches -∞ when $\Delta G_{r}^{'}\to0$ as the denominator of the first term of Eq. S33 approaches zero. Altogether, both reaction elasticities approach infinity close to equilibrium, supporting the idea that small changes in reactant concentrations have relative great impacts on the reaction rate.

$\lim_{\Delta G_{r}^{'}\to0} \varepsilon_{P}^{v}=\lim_{\Delta G_{r}^{'}\to0} -\frac{\exp\left(\Delta G_{r}^{'}/RT\right)}{1-\exp\left(\Delta G_{r}^{'}/RT\right)}-\frac{\exp\left(\Delta G_{r}^{'}/RT\right)\cdot K_{\text{eq}}K_{A}/K_{P}}{1+A/K_{A}+\exp\left(\Delta G_{r}^{'}/RT\right)\cdot K_{\text{eq}}K_{A}/K_{P}}$ (S33)

- 1. Far from equilibrium elasticity analysis

We are now interested in determining the elasticities’ behaviour far from equilibrium when $\Delta G_{r}^{'}\to-\infty$. As before, the sought limits for the reaction elasticities are given by,

$\begin{matrix} \lim_{\Delta G_{r}^{'}\to-\infty} \varepsilon_{S}^{v}=\lim_{\Delta G_{r}^{'}\to-\infty} \frac{1}{1-\exp\left(\Delta G_{r}^{'}/RT\right)}-\frac{A/K_{A}}{1+A/K_{A}+\exp\left(\Delta G_{r}^{'}/RT\right)\cdot K_{\text{eq}}K_{A}/K_{P}} \\ \lim_{\Delta G_{r}^{'}\to-\infty} \varepsilon_{P}^{v}=\lim_{\Delta G_{r}^{'}\to-\infty} -\frac{\exp\left(\Delta G_{r}^{'}/RT\right)}{1-\exp\left(\Delta G_{r}^{'}/RT\right)}-\frac{\exp\left(\Delta G_{r}^{'}/RT\right)\cdot K_{\text{eq}}K_{A}/K_{P}}{1+A/K_{A}+\exp\left(\Delta G_{r}^{'}/RT\right)\cdot K_{\text{eq}}K_{A}/K_{P}} \end{matrix}$ (S34)

As $\Delta G_{r}^{'}\to-\infty,\text{ exp(}\Delta G_{r}^{'}/RT\text{)}\to0$and hence the product elasticity approaches to 0. On the contrary, the substrate elasticity is non-zero and approaches to the following expression,

$\lim_{\Delta G_{r}^{'}\to-\infty} \varepsilon_{S}^{v}=1-\frac{A/K_{A}}{1+A/K_{A}}=\frac{1}{1+A/K_{A}}$ (S35)

Eq. S35 is equivalent to the substrate elasticity for a Uni-Uni irreversible Michealis-Menten kinetics [[8](#_ENREF_8)]. Notably, this expression is consistent with the observation that strongly saturated enzymes, *i.e.* *A*/*K_A_* high, have substrate elasticity close to zero. Nevertheless, this expression also suggests that it is not a necessary condition to have a highly saturated enzyme to have a thermodynamically favoured reaction. Indeed, extremely negative $\Delta G_{r}^{\circ}$ and/or low product concentrations can also drive the reaction. As such, the substrate elasticity can be non-zero even for a thermodynamically favoured reaction as long as Eq. S30 and the respective Haldane relationships are fulfilled [[9](#_ENREF_9)].

1. Calculation of Hill curves

The Hill plot is given by the slope of the normal binding curve weighted by the amount of ligand bound according to the expression,

$n_{\text{H}}=\frac{d\bar{X}/d\ln x}{\bar{X}\left(1-\bar{X}/n\right)}$ (S36)

where $\bar{X}$ is the ligand bound, *x* represents the ligand concentration and *n* denotes the number of binding sites. In the case of the MWC model, this parameter is closely approximated by a function of the binding polynomial (*P*) and the number of binding sites [[10](#_ENREF_10)],

$n_{\text{H}}=n\cdot\frac{PP''-\left(P'\right)^{2}}{P'\left(nP-P'\right)}$ (S37)

Notably, the binding polynomial is a function of the allosteric equilibrium constant (*L*_0_) and the kinetic parameters of the R and T states. Within the MWC setting, the allosteric effectors modify the allosteric constant *L*_0_ yielding an apparent allosteric constant $L_{0}^{'}$ [[1](#_ENREF_1)],

$L_{0}^{'}=L_{0}\left(\frac{1+I/K_{I}}{1+A/K_{A}}\right)^{n}$ (S38)

where *I/K_I_* and *A/K_A_* represent an allosteric inhibitor and activator weighted by their respective dissociation constants. By modifying the allosteric constant accordingly, the MWC model can capture the effect of the allosteric effectors on the kinetics of the oligomeric enzyme.

References

1. Monod J, Wyman J, Changeux JP (1965) On the nature of allosteric transitions: a plausible model. J Mol Biol 12: 88-118.

2. Geng Y, Bush M, Mosyak L, Wang F, Fan QR (2013) Structural mechanism of ligand activation in human GABA(B) receptor. Nature 504: 254-259.

3. Milligan G, Smith NJ (2007) Allosteric modulation of heterodimeric G-protein-coupled receptors. Trends Pharmacol Sci 28: 615-620.

4. Trankle C, Dittmann A, Schulz U, Weyand O, Buller S, et al. (2005) Atypical muscarinic allosteric modulation: Cooperativity between modulators and their atypical binding topology in muscarinic M-2 and M-2/M-5 chimeric receptors. Mol Pharmacol 68: 1597-1610.

5. Colquhoun D, Dowsland Ka, Beato M, Plested AJR (2004) How to impose microscopic reversibility in complex reaction mechanisms. Biophys J 86: 3510-3518.

6. Tran LM, Rizk ML, Liao JC (2008) Ensemble modeling of metabolic networks. Biophys J 95: 5606-5617.

7. Leskovac V (2003) Comprehensive Enzyme Kinetics: Springer Science & Business Media.

8. Cornish-Bowden A (2012) Fundamentals of Enzyme Kinetics: Wiley-Blackwell.

9. Alberty R (2006) Relations between biochemical thermodynamics and biochemical kinetics. Biophys Chem 124: 11-17.

10. Wyman J, Gill SJ (1990) Binding and Linkage Functional Chemistry of Biological Macromolecules. Mill Valley, California: University Science Books.
